# Pathological hydrogen peroxide triggers the fibrillization of wild-type SOD1 via sulfenic acid modification of Cys-111

**DOI:** 10.1038/s41419-017-0106-4

**Published:** 2018-01-22

**Authors:** Wen-Chang Xu, Jin-Zhao Liang, Cheng Li, Zhi-Xin He, Han-Ye Yuan, Ben-Yan Huang, Xiao-Ling Liu, Bo Tang, Dai-Wen Pang, Hai-Ning Du, Yi Yang, Jie Chen, Lei Wang, Min Zhang, Yi Liang

**Affiliations:** 1 State Key Laboratory of Virology, College of Life Sciences, Wuhan University, Wuhan, 430072 China; 2 National Laboratory of Biomacromolecules, CAS Center for Excellence in Biomacromolecules, Institute of Biophysics, Chinese Academy of Sciences, Beijing, 100101 China; 3 University of Chinese Academy of Sciences, Beijing, 100049 China; 4 Department of Neurology Tongji Hospital, Tongji Medical College, Huazhong University of Science and Technology, Wuhan, 430030 China; 5 College of Chemistry and Molecular Sciences, State Key Laboratory of Virology, Wuhan University, Wuhan, 430072 China; 6 Hubei Key Laboratory of Cell Homeostasis, College of Life Sciences, Wuhan University, Wuhan, 430072 China; 7 State Key Laboratory of Bioreactor Engineering, School of Pharmacy, East China University of Science and Technology, Shanghai, 200237 China

## Abstract

Amyotrophic lateral sclerosis (ALS) involves the abnormal posttranslational modifications and fibrillization of copper, zinc superoxide dismutase (SOD1) and TDP-43. However, how SOD1-catalyzed reaction product hydrogen peroxide affects amyloid formation of SOD1 and TDP-43 remains elusory. 90% of ALS cases are sporadic and the remaining cases are familial ALS. In this paper, we demonstrate that H_2_O_2_ at pathological concentrations triggers the fibrillization of wild-type SOD1 both in vitro and in SH-SY5Y cells. Using an anti-dimedone antibody that detects sulfenic acid modification of proteins, we found that Cys-111 in wild-type SOD1 is oxidized to C-SOH by pathological concentration of H_2_O_2_, followed by the formation of sulfenic acid modified SOD1 oligomers. Furthermore, we show that such SOD1 oligomers propagate in a prion-like manner, and not only drive wild-type SOD1 to form fibrils in the cytoplasm but also induce cytoplasm mislocalization and the subsequent fibrillization of wild-type TDP-43, thereby inducing apoptosis of living cells. Thus, we propose that H_2_O_2_ at pathological concentrations triggers the fibrillization of wild-type SOD1 and subsequently induces SOD1 toxicity and TDP-43 toxicity in neuronal cells via sulfenic acid modification of Cys-111 in SOD1. Our Western blot and ELISA data demonstrate that sulfenic acid modified wild-type SOD1 level in cerebrospinal fluid of 15 sporadic ALS patients is significantly increased compared with 6 age-matched control patients. These findings can explain how H_2_O_2_ at pathologic concentrations regulates the misfolding and toxicity of SOD1 and TDP-43 associated with ALS, and suggest that sulfenic acid modification of wild-type SOD1 should play pivotal roles in the pathogenesis of sporadic ALS.

## Introduction

The abnormal post-translational modifications and misfolding of human SOD1 and TDP-43 in motor neuron cells play a crucial role in the etiology of amyotrophic lateral sclerosis (ALS)^1–11^. Ninety percent of ALS cases are sporadic^1,3^; however, little is known about the mechanism underlying most sporadic ALS and the reason why ALS and frontotemporal lobar degeneration (FTLD) are sometimes overlapping^8^. Pathologically, SOD1 is the major composition of inclusions found in sporadic ALS patient’s spinal cord^3,12^, and TDP-43 is the main composition of ubiquitin-positive inclusions observed in ALS and FTLD patients' brain and spinal cord^10,11,13^. The misfolding of SOD1 and TDP-43 has been widely studied during the past 20 years^2–7,10,11,14–25^. The characterization of factors regulating such misfolding is crucial to illuminate the pathology of ALS and FTLD and to help set up medical treatment.

SOD1 is essential for H_2_O_2_ induced oxidative stress during cell signaling^26,27^. Though H_2_O_2_ concentration inside cells is usually very low under physiological conditions, it can increase up to 150 μM under pathological oxidative conditions^26,28–32^. It has been demonstrated that an iper-oxidized form of wild-type SOD1 with toxic properties exist not only in sporadic ALS patient-derived lymphoblasts, but also in healthy control lymphoblasts treated with H_2_O_2_ at a pathological concentration^17^. However, how H_2_O_2_ at pathological concentrations (10–100 μM)^17,29^, a product of SOD1-catalyzed reaction^9^, regulates the misfolding and toxicity of wild-type SOD1 and TDP-43 in neuronal cells, associated with sporadic ALS and FTLD, remains elusory.

In this study, we used pathological concentration of H_2_O_2_ to trigger the oligomerization and fibrillization of wild-type human SOD1. Our results indicate that pathological H_2_O_2_ did trigger the fibrillization of wild-type SOD1 via sulfenic acid modification of Cys-111 (C-SOH) in this enzyme in living neuronal cells, accompanied by cytoplasm mislocalization and fibrillization of wild-type human TDP-43, thereby inducing neuronal apoptosis. What is more is that we observed a significant increase of sulfenic acid-modified wild-type SOD1 level in cerebrospinal fluid (CSF) of sporadic ALS patients compared with age-matched controls. Our findings link SOD1/TDP-43 misfolding and disease-causing functions regulated by pathological H_2_O_2_ to the pathology of sporadic ALS and FTLD.

## Results

### Pathological concentration of hydrogen peroxide triggers SOD1 fibrillization

**Fig. 1:**
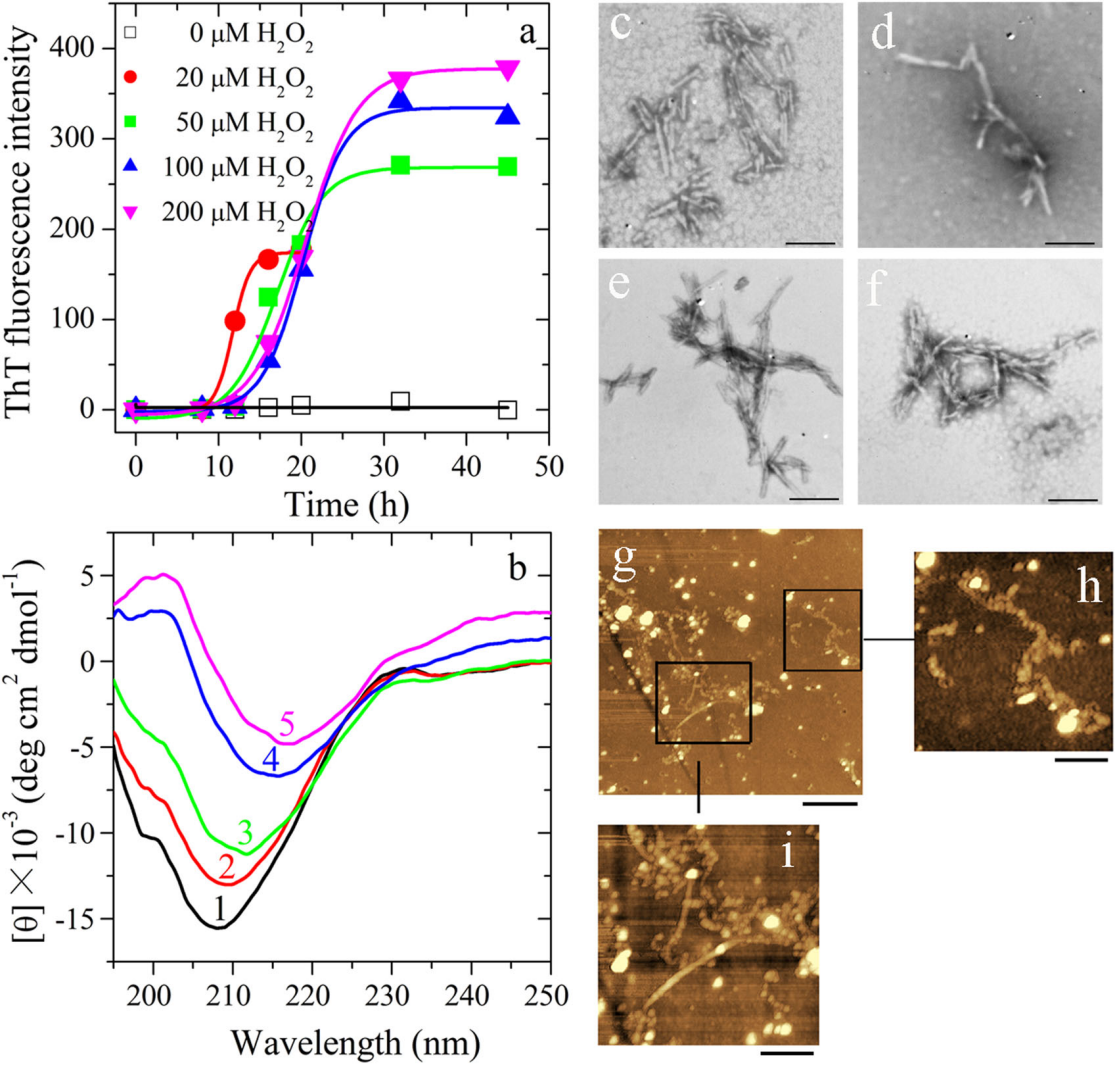
Hydrogen peroxide at pathological concentrations triggers the fibrillization of wild-type human SOD1 in vitro Thirty micro mole apo wild-type SOD1 (**a**) was treated without or with 20–200 μM H_2_O_2_ in 20 mM Tris-HCl buffer (pH 7.4) and at 37 °C with constant agitation for up to 45 h and analyzed by ThT binding assays (**a**), or for 45 h (curves 1 and 3–5) or 20 h (curve 2) and analyzed by CD spectroscopy (**b**). The H_2_O_2_ concentrations were 0 μM (open square in (**a**) or curve 1 in (**b**)), 20 μM (solid circle in (**a**) or curve 2 in (**b**)), 50 μM (solid square in (**a**) or curve 3 in (**b**)), 100 μM (solid up triangle in (**a**) or curve 4 in (**b**)), and 200 μM (solid down triangle in (**a**) or curve 5 in (**b**)), respectively. Solid lines show the best sigmoidal fit for the ThT intensity-time curves (**a**). Negative-stain transmission electron micrographs (**c**–**f**) and AFM images (**g**–**i**) of aggregates produced from 30 μM apo wild-type SOD1 incubated with 20-200 μM H_2_O_2_ (**c**, 20 μM; **d**, 50 μM; **e**, **g**–**i**, 100 μM; and **f**, 200 μM) at 37 °C for 45 h or 20 h (**e**). The enlarged regions (**h**) and (**i**) show two-fold and four-fold enlarged images from (**g**), respectively, and display the detailed structure of SOD1 fibrils. The scale bars are 200 nm (**c–f**), 2 μm (**g**), 1 μm (**h**), and 500 nm (**i**), respectively

As shown in Fig. 1a, at pH 7.4, apo wild-type SOD1 (apo-SOD1) did form fibrils when treated with 20, 50, 100, or 200 μM H_2_O_2_, but did not form fibrils when treated without H_2_O_2_ (Fig. 1a). Interestingly, we found that an increasing concentration of H_2_O_2_ from 20 to 200 μM increased the amount of apo-SOD1 filaments by remarkably enhancing the maximum ThT fluorescence intensity, but dramatically decelerated the fibrillization of apo-SOD1 by elongating the lag time to a great extent (from 9.48 ± 0.60 to 14.6 ± 0.8 h), indicating a delay in the nucleation phase (Fig. 1a). The fibrillization of apo-SOD1 induced by 20–200 μM H_2_O_2_ was further confirmed by CD spectroscopy, TEM, and AFM^33–35^. As seen from Fig. 1b, in the absence of H_2_O_2_, the CD spectrum measured for apo-SOD1 had a weakly positive band at 230 nm and a strong negative peak at 208 nm, which reflects the antiparallel β-strand architecture of apo-SOD1^36^. With the increase of H_2_O_2_ concentration from 20 to 200 μM, the positive peak at 230 nm of apo-SOD1 disappeared gradually and the negative peak of apo-SOD1 gradually moved into 216 nm (Fig. 1b), indicating that apo-SOD1 formed amyloid fibrils with β-sheet-rich conformation under such conditions. TEM images indicate that an increasing concentration of H_2_O_2_ from 20 to 200 μM did not have significant effect on the morphology of apo-SOD1 aggregates (Fig. 1c–f). The fibrils of apo-SOD1 appear twisted and with a branched structure with a length of 100–300 nm under all conditions (Fig. 1c–f). However, similar to those previously reported^30^, H_2_O_2_ at high concentrations induced non-amyloid aggregation of apo-SOD1 (Figure S1a, b). Some long amyloid fibrils (Fig. 1g, i) and some beaded amyloid fibrils (Fig. 1g, h) were also observed using AFM when apo-SOD1 was treated with 100 μM H_2_O_2_. Clearly, apo-SOD1 did form fibrils when treated with 20–200 μM H_2_O_2_ (Fig. 1a–i), but did not form fibrils when treated without H_2_O_2_ (Fig. 1a, b) or with 1.0–2.0 mM H_2_O_2_ (Figure S1a,b). Therefore, it is pathological H_2_O_2_ that triggered wild-type SOD1 fibrillization.

**Fig. 2:**
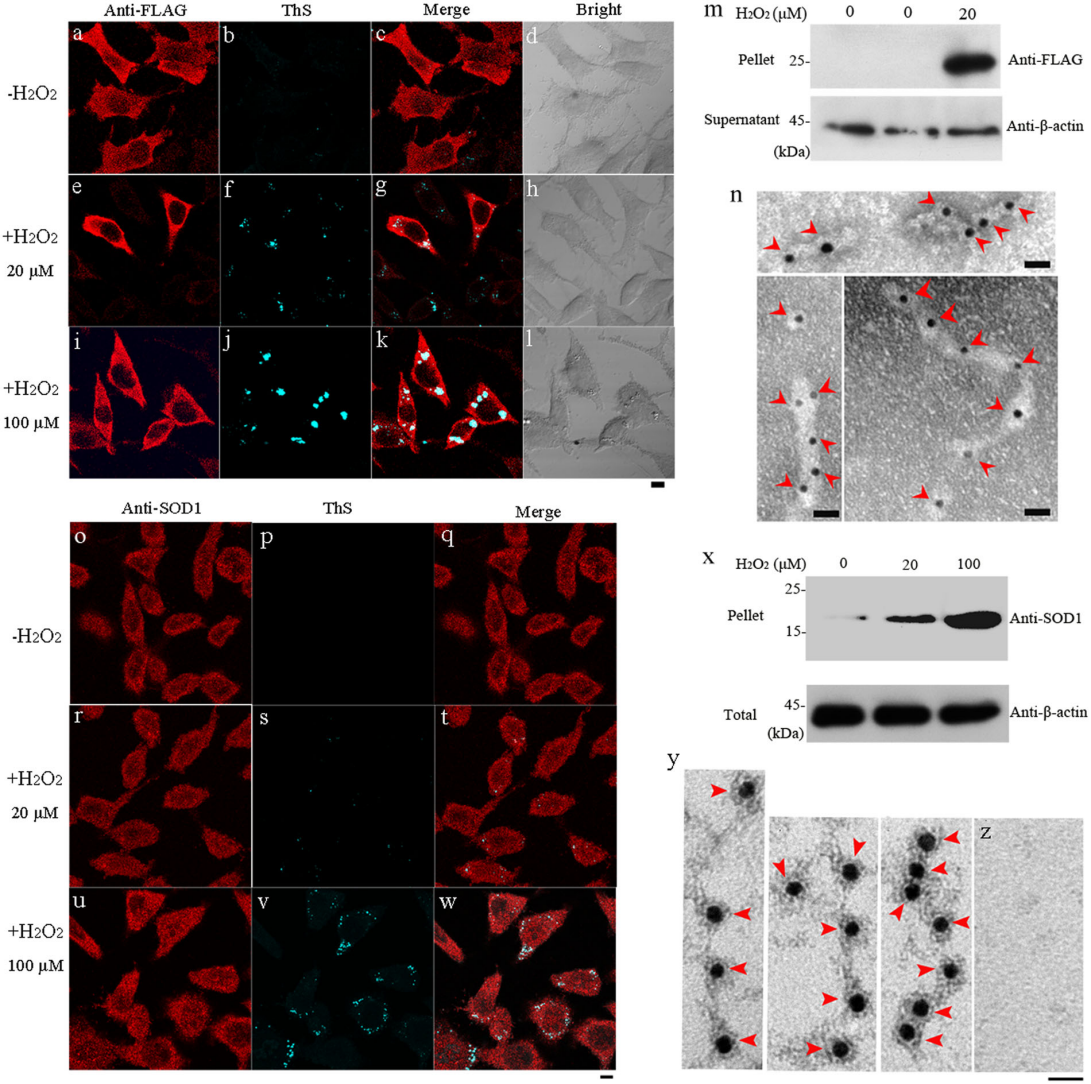
Hydrogen peroxide at pathological concentrations triggers the fibrillization of wild-type human SOD1 in SH-SY5Y cells SOD1 stable cells treated with 20 (+) (**e–h**) or 100 μM (+) (**i–l**) H_2_O_2_, and SH-SY5Y cells treated with 20 (+) (**r–t**) or 100 μM (+) (**u–w**) H_2_O_2_, compared with SOD1 stable cells and SH-SY5Y cells treated without H_2_O_2_ (−) (**a**–**c**, **o**–**q**). SOD1 stable cells (**a**–**l**) and SH-SY5Y cells (**o**–**w**) were treated with 0–100 μM H_2_O_2_ at 37 °C for 4 and 6 days, respectively; fixed, ruptured and stained by thioflavin S (green), subsequently immunostained by mouse anti-FLAG antibody (**a**, **e**, **i**) or rabbit anti-SOD1 antibody (**o**, **r**, **u**) and homologous IgG conjugated with Alexa Fluo-546 (red), and observed with confocal microscopy. SOD1 stable cells (**m**) and SH-SY5Y cells (**x**) were treated at 37 °C with 0–20 μM H_2_O_2_ for 4 days and 0–100 μM H_2_O_2_ for 6 days, respectively, and then detected with western blotting. The Sarkosyl-insoluble pellets from SOD1 stable cells (**m**) and SH-SY5Y cells (**x**) were detected with mouse anti-FLAG antibody and rabbit anti-SOD1 antibody, respectively, and the supernatants from both cells were probed by mouse anti-β-actin antibody. H_2_O_2_ concentration from left to right (**m**) was zero (SH-SY5Y cells), zero (SOD1 stable cells), and 20 μM (SOD1 stable cells), respectively, and that from left to right (**x**) was zero (SH-SY5Y cells), 20 μM (SH-SY5Y cells), and 100 μM (SH-SY5Y cells), respectively. Untreated cells were the control (**m**, **x**, **z**). Aggregates extracted SOD1 stable cells (**n**) and SH-SY5Y cell line (**y**) incubated with 100 μM H_2_O_2_ were labeled by gold particles conjugated with anti-mouse/rabbit IgG. Red arrowheads were used to highlight amyloid fibrils. SOD1 fibrils were clearly observed by immunogold electron microscopy in both cases. Untreated SH-SY5Y cells were used as the control (**z**). The scale bars represent 10 μm (**a**–**l**),(**o**–**w**) and 20 nm (**n**, **y**, **z**), respectively

We then studied the effect of H_2_O_2_ treatment on wild-type SOD1 fibrillization in SH-SY5Y cells. As shown in Fig. 2, when treated with 20 (Fig. 2e–h, r–t) or 100 μM (Fig. 2i–l, u–w) H_2_O_2_, not only wild-type SOD1 formed fibrils in SOD1 stable cells but also endogenous SOD1 formed fibrils in cytoplasm of SH-SY5Y cells. These were detected with anti-FLAG antibody (red) or anti-SOD1 antibody (red) and ThS staining (green) (Fig. 2a, b, e, f, i, j, o, p, r, s, u, v). The merge image (white or light gray) proved that stably expressed wild-type SOD1 or endogenous SOD1 was co-localized with ThS-positive amyloids (Fig. 2c, g, k, q, t, w). As seen from Fig. 2, wild-type SOD1 formed amyloid fibrils in SOD1 stable cells when treated with 20 μM H_2_O_2_ (m), and endogenous SOD1 formed fibrils in SH-SY5Y cells when treated with 20 or 100 μM H_2_O_2_ (x), as detected by Western blot. Furthermore, an increasing concentration of H_2_O_2_ from 20 (Fig. 2 f, g, s, t, x) to 100 μM (Fig. 2j,k,v,w,x) resulted in a remarkable increase in the amount of SOD1 fibrils, as indicated by the growing intensity of white or light gray spots or by the growing densities of SOD1 fibril bands. Gold particles were used to label the aggregates extracted from SOD1 stable cells and SH-SY5Y cell line incubated with 100 μM H_2_O_2_, and SOD1 fibrils were clearly observed using immunogold electron microscopy^37,38^ in both cases (Fig. 2n, y). Taken together, both stably expressed wild-type SOD1 and endogenous SOD1 formed amyloid fibrils in cytoplasm of SH-SY5Y cells treated with 20 μM (Fig. 2e–h, m, r–t) or 100 μM (Fig. 2i–l, n, u–w, x, y) H_2_O_2_, but did not form amyloid fibrils when treated without H_2_O_2_ (Fig. 2a–c, o–q, m, x, z). Therefore, pathological H_2_O_2_ did trigger fibril formation of wild-type SOD1 in neuronal cells.

### Cys-111 in wild-type SOD1 is oxidized to C-SOH by pathological H_2_O_2_

H_2_O_2_ at high concentrations (≥1 mM) induces non-amyloid aggregation of apo-SOD1^30^ by over-oxidizing Cys-111 in this protein to C-SO_2_H and C-SO_3_H^12,31^, which was confirmed by Figures S1 and S2. However, as mentioned above, pathological H_2_O_2_ did trigger the fibrillization of wild-type SOD1. We thus wanted to know whether pathological concentration of H_2_O_2_ could oxidize Cys-111 in wild-type SOD1 differently from high concentrations of H_2_O_2_, thereby inducing fibril formation of this protein.

**Fig. 3:**
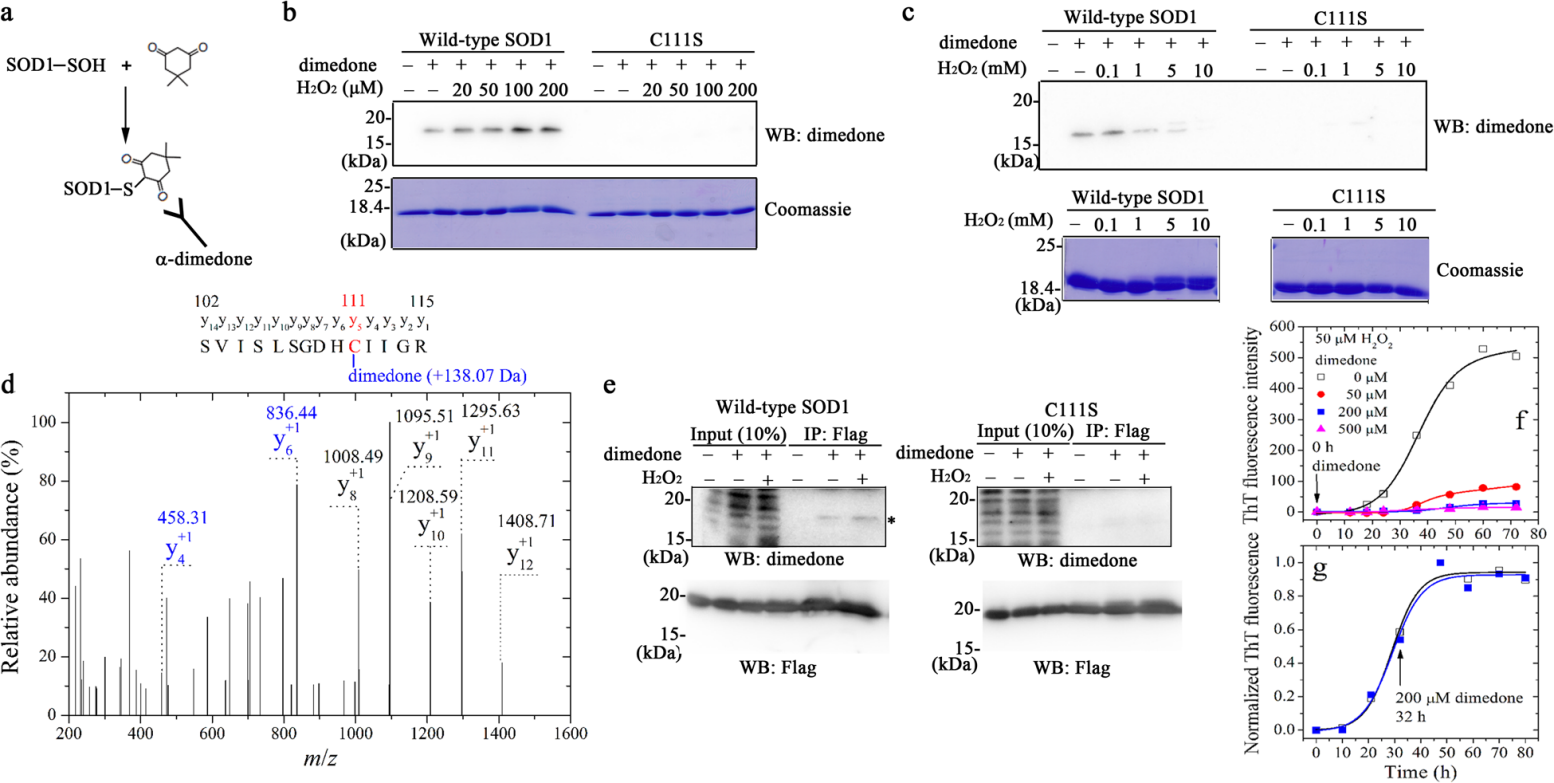
Cys-111 in wild-type human SOD1 is oxidized to C-SOH by pathological concentration of H_2_O_2_ Strategy for detecting sulfenic acid modification of SOD1 with an anti-dimedone antibody (**a**). Sulfenic acid modification of wild-type SOD1 was observed when treated with 0-200 μM H_2_O_2_ in vitro (WB, **b**) or 100 μM H_2_O_2_ in cells (asterisk, WB: dimedone, **e**), but that of C111S was not observed when treated with 0–200 μM H_2_O_2_ in vitro (WB, **b**) or 100 μM H_2_O_2_ in cells (WB: dimedone, **e**). 10 μM apo wild-type SOD1 and its cysteine mutant C111S were treated with 0–200 μM H_2_O_2_ (**b**) or 0–10 mM H_2_O_2_ (**c**) for 30 min at 25 °C, blocked with (+) or without (-) 5 mM dimedone, and then detected by Western blot using anti-dimedone antibody (WB, **b**, **c**). Coomassie blue staining of these protein samples was set as a loading control (Coomassie, **b**, **c**). H_2_O_2_ concentration from left to right was zero (−), zero (−), 20, 50, 100, and 200 μM (**b**) or zero (−), 0.1, 1.0, 5.0, and 10.0 mM (**c**), respectively. Over-oxidized forms of wild-type SOD1 (upper shifted band), SOD1-SO_2_H/-SO_3_H, were observed in vitro when treated with 1.0–10.0 mM H_2_O_2_ for 30 min (**c**). In contrast, no oxidized band was observed for C111S under the same conditions (**c**). The Coomassie Blue-stained gels of 10 μM apo wild-type SOD1 treated with 100 μM H_2_O_2_ for 30 min and blocked with 5 mM dimedone were scissored out, chopped, trypsinized, and then analyzed with LC-MS/MS. A MS^2^ analysis of dimedone-labeled parent peptide S^102^VISLSGDHCIIGR^115^ digested by trypsin (**d**). Analysis of the y-ions indicates the formation of dimedone adduct (+138.07 Da) (**d**) of sulfenic acid in Cys-111, demonstrating sulfenic acid modification of Cys-111 in human SOD1. HEK293T cells transiently overexpressing FLAG-tagged wild-type SOD1 or its C111S variant were incubated with (+) or without (−) 100 μM H_2_O_2_ for 4 days at 25 °C, and blocked with (+) or without (−) 5 mM dimedone (**e**). Cell lysates were immunoprecipitated with *α*-Flag M2 affinity gel and then detected by Western blot using anti-dimedone antibody (WB: dimedone, **e**) or anti-FLAG antibody (WB: Flag, **e**). 50–500 μM dimedone added at zero time almost blocked the fibrillization of wild-type SOD1 in vitro (**f**), but 200 μM dimedone added at 32 h had no obvious effects on the fibrillization (**g**). 30 μM apo wild-type SOD1 was treated with 50 μM H_2_O_2_, but without dimedone (open square in **f** and **g**), and then titrated with 50 μM dimedone (solid circle in **f**), 200 μM dimedone (solid square in **f**) or 500 μM dimedone (solid triangle in **f**) at 0 h (**f**), or 200 μM dimedone (solid square in **g**) at 32 h (**g**), respectively. Black arrows indicate the beginning of titrations. Solid lines show the best sigmoidal fit for the ThT intensity-time curves (**f**, **g**). ThT binding assays were carried out at 37 °C

In this study, we detected sulfenic acid modification of SOD1 by pathological concentration of H_2_O_2_ using an anti-dimedone antibody (Fig. 3a). Sulfenic acid modification to wild-type SOD1 was clearly observed when this enzyme was treated with 0–200 μM H_2_O_2_ in vitro for 30 min (Fig. 3b, the top left) or 0–100 μM H_2_O_2_ in cells for 2 h (Fig. 3e, the top left), but for single cysteine mutant C111S, sulfenic acid modification was not observed when incubated with 0–200 μM H_2_O_2_ in vitro (Fig. 3b, the top right) or 0–100 μM H_2_O_2_ in cells (Fig. 3e, the top right). To our surprise, an upper shifted band representing SOD1-SO_2_H and SOD1-SO_3_H was not observed for apo-SOD1 when treated with 0–200 μM H_2_O_2_ for 30 min (Fig. 3b, the bottom left). Interestingly, we found that an increasing concentration of H_2_O_2_ from 0 to 100 μM resulted in an increase in the amount of sulfenic acid-modified wild-type SOD1 by remarkably enhancing the density of the SOD1 band probed by anti-dimedone antibody (Fig. 3b, the top left). Further increasing the concentration of H_2_O_2_ up to 5.0 mM, however, strongly decreased the amount of sulfenic acid-modified wild-type SOD1 (Fig. 3b, c, the top left), and sulfenic acid modification was not observed when this enzyme was incubated with 10.0 mM H_2_O_2_ for 30 min (Fig. 3c, the top left). Analysis of the y-ions in Fig. 3d indicated the formation of dimedone adduct (+138.07 Da) of sulfenic acid in Cys-111, demonstrating sulfenation of Cys-111 in apo-SOD1 when treated with 100 μM H_2_O_2_ for 30 min. Clearly, Cys-111 in wild-type SOD1 was oxidized to C-SOH by pathological concentration of H_2_O_2_. Furthermore, 200/500 μM dimedone added at zero time (Fig. 3f) or 1.0 mM dimedone added at 6 h (Figure S3) almost blocked the fibrillization of apo-SOD1 triggered by 50 or 100 μM H_2_O_2_ via blocking sulfenic acid modification to SOD1 in the lag phase, the first stage of protein misfolding^39^. In other words, sulfenic acid-modified SOD1 oligomers were formed in the lag phase. In contrast, 200 μM dimedone added at 32 h in the growth phase did not have obvious effect on the fibrillization of apo-SOD1 induced by 50 μM H_2_O_2_ (Fig. 3g). Therefore, pathological H_2_O_2_ did trigger the fibrillization of wild-type SOD1 via sulfenation of Cys-111.

### Sulfenic acid-modified SOD1 oligomers induce the fibrillization of wild-type SOD1/TDP-43 in cells

**Fig. 4:**
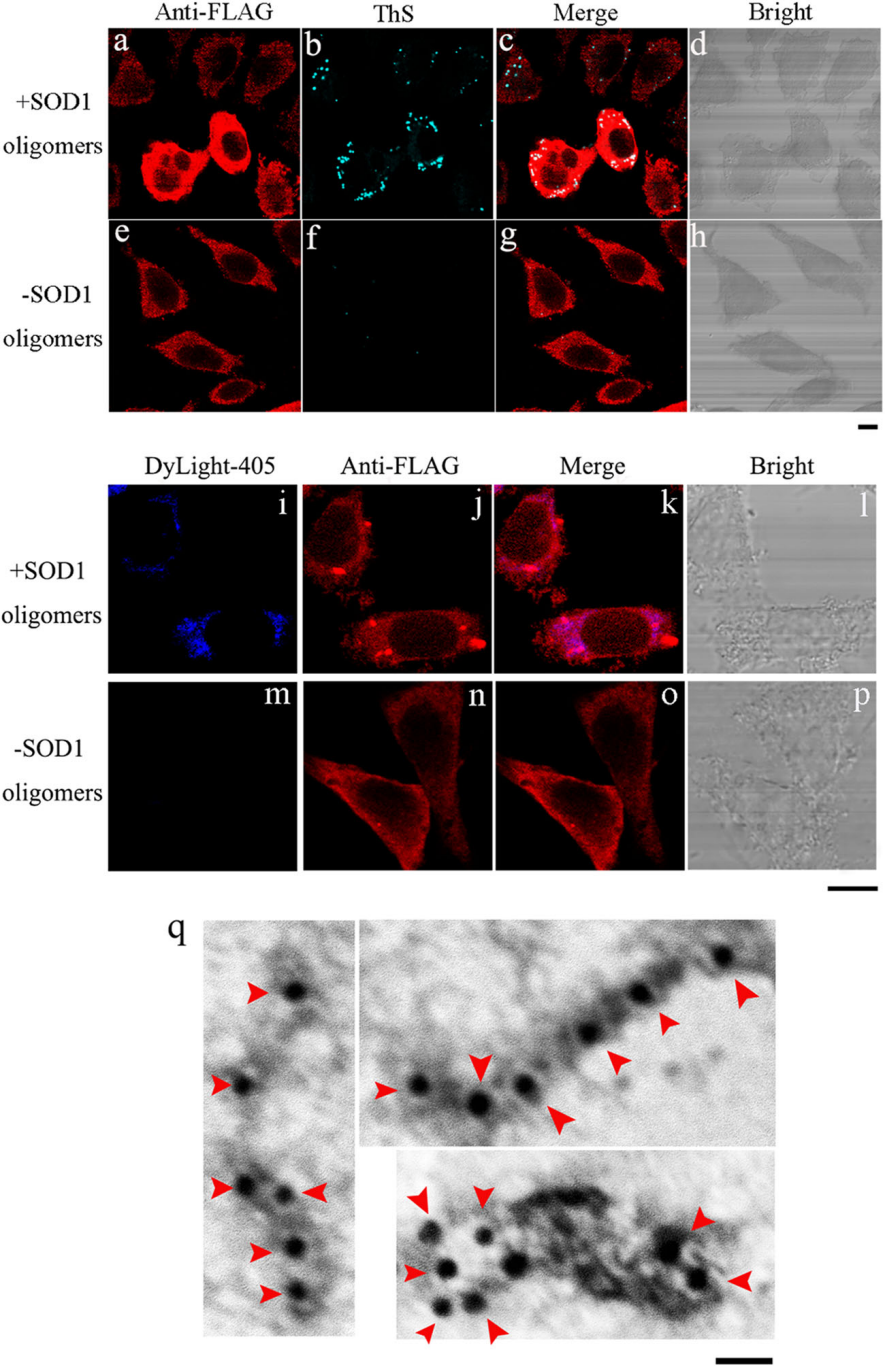
Sulfenic acid-modified SOD1 oligomers induce the fibrillization of human SOD1 in SH-SY5Y cells SOD1 stable cells treated with 5 μM sulfenic acid-modified SOD1 oligomers (+) (**a**–**d**), compared with SOD1 stable cells treated without SOD1 oligomers (−) (**e**–**h**). SOD1 stable cells (**a**–**h**) were incubated with 0 or 5 μM sulfenic acid-modified SOD1 oligomers for 3 days at 37 °C, fixed, ruptured, stained by thioflavin S (green), subsequently immunostained by anti-FLAG antibody and IgG conjugated with Alexa Fluo-546 (red), and observed with confocal microscopy. SOD1 stable cells treated with 1 μM biotinylated sulfenic acid-modified SOD1 oligomers (+) (**i**–**l**), compared with SOD1 stable cells when treated without SOD1 oligomers (−) (**m**–**p**). SOD1 stable cells (**i**–**p**) were incubated with 0 or 1 μM biotinylated sulfenic acid-modified SOD1 oligomers for 3 days at 37 °C, fixed, ruptured and detected by streptavidin DyLight-405 dye (blue), subsequently immunostained by anti-FLAG antibody and IgG conjugated with Alexa Fluo-546 (red); and observed with confocal microscopy. Aggregates extracted from SOD1 stable cells incubated with 5 μM sulfenic acid-modified SOD1 oligomers (**q**) for 3 days at 37 °C were labeled with gold particles. Red arrowheads were used to highlight amyloid fibrils. SOD1 fibrils were clearly observed by immunogold electron microscopy in such a case. Sulfenic acid-modified SOD1 oligomers (**a**–**d**) were formed when apo wild-type SOD1 (30 μM) was incubated with 100 μM hydrogen peroxide in 20 mM Tris-HCl buffer (pH 7.4) and at 37 °C for 6 h, and then labeled with biotin (**i**–**l**). The scale bars represent 10 μm (**a**–**p**) and 20 nm (**q**), respectively

In this study, we firstly obtained sulfenic acid-modified SOD1 oligomers in the lag phase by incubating 30 μM apo-SOD1 with 100 μM H_2_O_2_ for 6 h. We then studied the effects of such SOD1 oligomers on misfolding of wild-type SOD1 in SH-SY5Y cells. As shown in Fig. 4, when treated with 5 μM sulfenic acid-modified SOD1 oligomers (Fig. 4a–d), stably expressed wild-type SOD1 or exogenous SOD1 oligomers entering into SOD1 stable cells formed fibrils in cytoplasm of SOD1 stable cells, as probed by anti-FLAG antibody (red) and ThS staining (green). The merge image (white or light gray) proved that wild-type SOD1 was co-localized with ThS-positive amyloids (Fig. 4c). We further proved that both stably expressed wild-type SOD1 and endogenous SOD1 did form fibrils (red spots) in cytoplasm of SOD1 stable cells treated with 1 μM biotinylated sulfenic acid-modified SOD1 oligomers (Fig. 4i–l and S4a–d). As shown in Fig. 4i, k and S4a, c, such SOD1 oligomers, stained by DyLight-405 (blue), entered into SH-SY5Y cells through endocytosis or macropinocytosis^15,23,40^ and were mainly located in the cytoplasm. As shown in Fig. 4i–l and S4a–d, when treated with 1 μM biotinylated SOD1 oligomers, not only blue spots but also red spots were observed, demonstrating that both stably expressed wild-type SOD1 and endogenous SOD1 formed fibrils in cytoplasm of SOD1 stable cells, as probed with anti-FLAG antibody (red) or anti-SOD1 antibody (red) and streptavidin DyLight-405 dye (blue). As shown in Figure S5, endogenous SOD1 formed Sarkosyl-insoluble fibrils when SH-SY5Y cells were treated with 5 μM sulfenic acid-modified SOD1 oligomers (Fig. 4a-c), as detected by Western blot. However, both stably expressed wild-type SOD1 and endogenous SOD1 did not form amyloid fibrils when cells were not treated with SOD1 oligomers (Fig. 4e–h, m–p, S4e–h, and S5a–c). Gold particles were used to label the aggregates extracted from SOD1 stable cells incubated with 5 μM SOD1 oligomers, and SOD1 fibrils were clearly observed in such a case (Fig. 4q). Therefore, sulfenic acid-modified SOD1 oligomers did propagate in a prion-like manner and induce SOD1 fibrillization in neuronal cells.

**Fig. 5:**
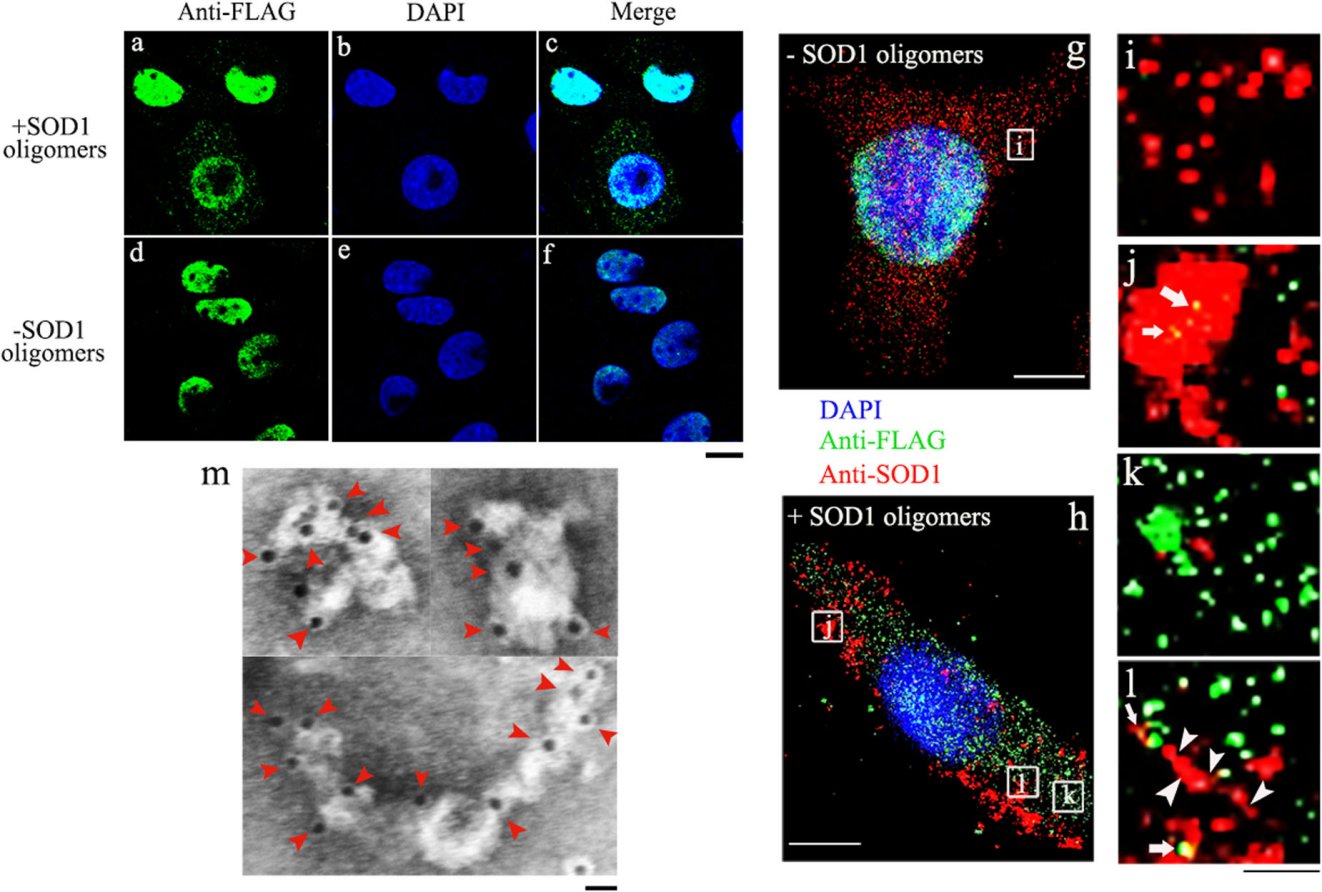
Sulfenic acid-modified SOD1 oligomers induce cytoplasm mislocalization of human TDP-43, the subsequent fibrillization of human TDP-43 and human SOD1 in SH-SY5Y cells TDP-43 stable cells treated with 5 μM sulfenic acid-modified SOD1 oligomers (+) (**a**-**c**, **h**, **k**), compared with TDP-43 stable cells treated without SOD1 oligomers (-) (**d**–**g**). The merge image (**h**, **j**, **l**, yellow dots) demonstrated co-localization of SOD1 aggregates (red), highlighted by using white arrowheads, with TDP-43 aggregates (green) in cytoplasm of TDP-43 stable cells. TDP-43 stable cells were incubated with 0 or 5 μM sulfenic acid-modified SOD1 oligomers for 3 days at 37 °C, fixed, ruptured, subsequently immunostained by mouse anti-FLAG antibody and IgG conjugated with Alexa Fluo-488 (green); and observed with confocal microscopy (**a**–**f**), or subsequently immunostained by mouse anti-FLAG antibody and goat anti-mouse IgG conjugated with Alexa Fluo-488 (green), and subsequently immunostained by rabbit anti-SOD1 antibody and donkey anti-rabbit IgG conjugated with Alexa Fluo-546 (red), and observed with super-resolution fluorescence microscopy (**g**–**l**). Nuclei were visualized by DAPI (blue). The enlarged regions (**i**) and (**j**–**l**) show ten-fold enlarged images from (**g**) and (**h**), respectively. Aggregates extracted from TDP-43 stable cells incubated with 5 μM sulfenic acid-modified SOD1 oligomers (**m**) for 3 days at 37 °C were labeled by gold particles. Red arrowheads were used to highlight amyloid fibrils. TDP-43 fibrils were clearly observed by immunogold electron microscopy in such a case. The scale bars represent 10 μm (**a**–**h**), 1 μm (**i–l**), and 20 nm (**m**), respectively

Thirdly, we studied the effects of sulfenic acid-modified SOD1 oligomers on misfolding of wild-type TDP-43 in SH-SY5Y cells. As shown in Fig. 5, when treated with 5 μM sulfenic acid-modified SOD1 oligomers (Fig 5a–c, h, k), TDP-43 was partly mislocalized from the nucleus to the cytosol of TDP-43 stable cells. However, stably expressed TDP-43 was correctly localized in the nuclei treated without SOD1 oligomers (Fig. 5d–f, g). Intracellular inclusions consisting of aggregates formed by either stably expressed wild-type TDP-43 (green spots in Fig. 5k) or SOD1 (red spots in Fig. 5j) were observed in TDP-43 stable cells, and the merge image (Fig. 5h, j, l; yellow dots) demonstrated co-localization of SOD1 aggregates (red), highlighted by using white arrowheads, with TDP-43 aggregates in the cytoplasm. Gold particles were used to label the aggregates extracted from TDP-43 stable cells incubated with 5 μM SOD1 oligomers, and TDP-43 fibrils were clearly observed in such a case (Fig. 5m). Therefore, sulfenic acid-modified SOD1 oligomers did propagate in a prion-like manner and induce cytoplasm mislocalization and the subsequent fibrillization of TDP-43 in neuronal cells.

### Sulfenic acid-modified SOD1 oligomers induce apoptosis of living cells via kindling SOD1/TDP-43 fibrillization

**Fig. 6:**
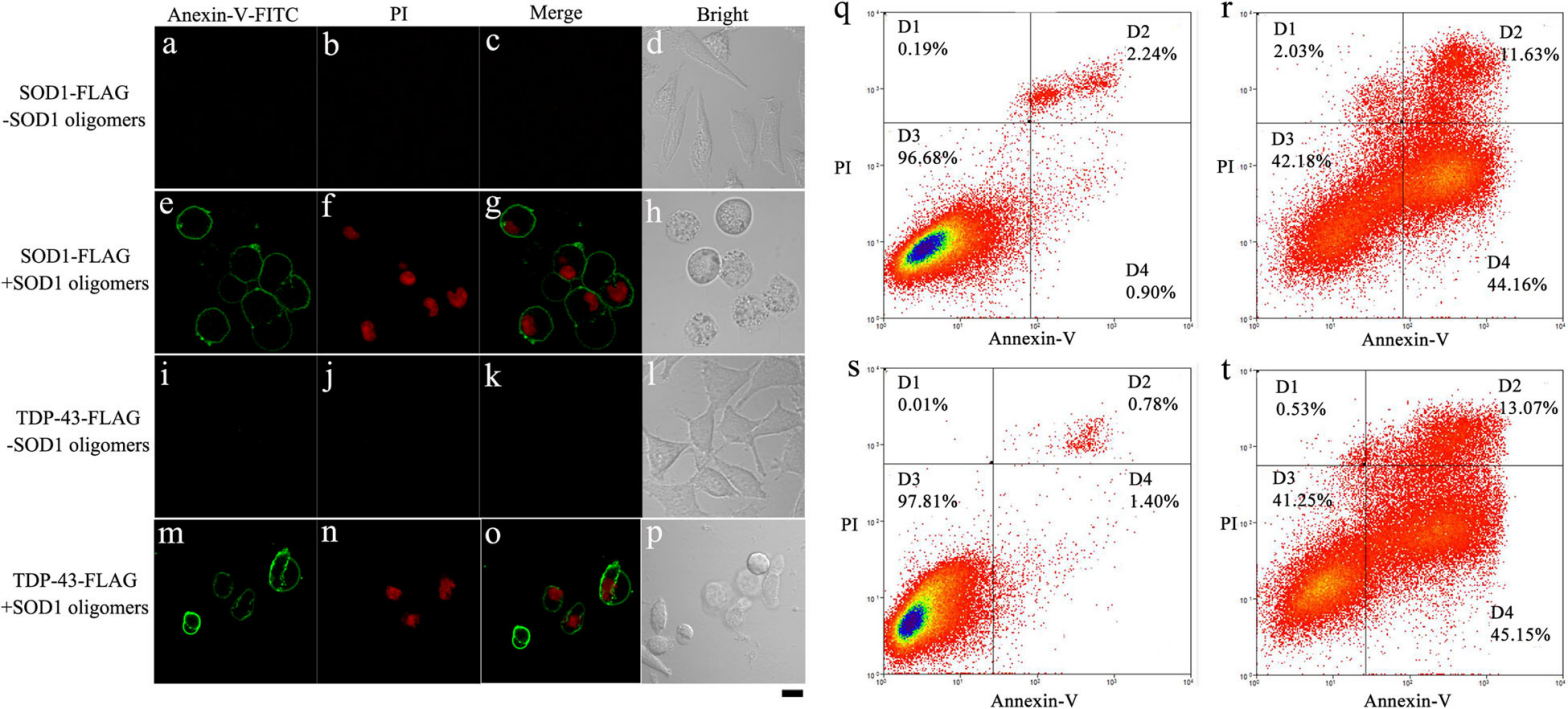
Sulfenic acid-modified SOD1 oligomers induce apoptosis of living SH-SY5Y cells Treatment of 5 μM sulfenic acid-modified SOD1 oligomers (+) (**e**–**h**) for 3 days did induce apoptosis of living SOD1 stable cells, but no apoptosis of living SOD1 stable cells was observed when treated without SOD1 oligomers (-) (**a**–**d**). Similarly, treatment of 5 μM sulfenic acid-modified SOD1 oligomers (+) (**m**–**p**) for 3 days did induce early and late apoptosis of living TDP-43 stable cells, but no apoptosis of living TDP-43 stable cells was observed when treated without SOD1 oligomers (-) (**i**–**l**). Annexin V-FITC (green) and PI (red) were used to double stain living SOD1/TDP-43 stable cells. Confocal laser scanning microscopy was used to observe apoptotic cells. The scale bar represents 10 μm (**a**–**p**). Living SOD1 stable cells (**r**) or TDP-43 stable cells (**t**) incubated with 10 μM sulfenic acid-modified SOD1 oligomers for 3 days showed much higher rates of apoptosis than those for SOD1 stable cells (**q**) or TDP-43 stable cells (**s**) incubated with 0 μM SOD1 oligomers. The percentage of apoptotic cells was determined by flow cytometry. Four quadrants divided by annexin V-FITC/PI staining were viable cells (D3 quadrant in **q**–**t**), early apoptotic cells (D4 quadrant in **q**–**t**), late apoptotic cells (D2 quadrant in **q**–**t**), and operation damaged cells (D1 quadrant in **q**–**t**), respectively

As mentioned above, both SOD1 and TDP-43 formed amyloid fibrils in cells induced by sulfenic acid-modified SOD1 oligomers, we wonder whether such fibrillization could induce apoptosis of living cells. As seen from Fig. 6e–h, treatment of 5 μM sulfenic acid-modified SOD1 oligomers for 3 days not only caused early apoptosis of some living SOD1 stable cells, as marked by FITC (green)-conjugated annexin V binding to phosphatidylserine exposed on the outer cellular membrane, but without propidium iodide (PI)^37,38,41,42^ (red) staining for nuclei, but also induced late apoptosis or necrosis of some other living SOD1 stable cells, as marked by FITC (green)-conjugated annexin V binding to phosphatidylserine exposed on the outer cellular membrane with PI (red) staining for nuclei. Similarly, treatment of 5 μM SOD1 oligomers for 3 days induced not only early apoptosis of some living TDP-43 stable cells (green outer membrane without red nucleus), but also late apoptosis or necrosis of some other living SOD1 stable cells (green outer membrane with red nucleus) (Fig. 6m–p). In contrast, such early and late apoptosis or necrosis was not observed in living cells stably expressing either FLAG-tagged SOD1 (Fig. 6a–d) or FLAG-tagged TDP-43 (Fig. 6i–l) when treated without SOD1 oligomers. As seen from Fig. 6, the percentages of early and late apoptotic cells in living SOD1 stable cells treated with 10 μM SOD1 oligomers for 3 days were 44.16% and 11.63%, respectively (r), much greater than those in SOD1 stable cells not incubated with SOD1 oligomers (Fig. 6q, 0.90% and 2.24%, respectively). Similarly, the percentages of early and late apoptotic cells in living TDP-43 stable cells incubated with 10 μM SOD1 oligomers for 3 days were 45.15% and 13.07%, respectively (Fig. 6t), much greater than those in TDP-43 stable cells not incubated with SOD1 oligomers (Fig. 6s, 1.40% and 0.78%, respectively). Clearly, sulfenic acid-modified SOD1 oligomers did induce apoptosis of living cells via kindling the fibrillization of SOD1 and TDP-43 in cells.

### Sulfenic acid-modified wild-type SOD1 level in CSF of sporadic ALS patients is significantly increased

**Fig. 7:**
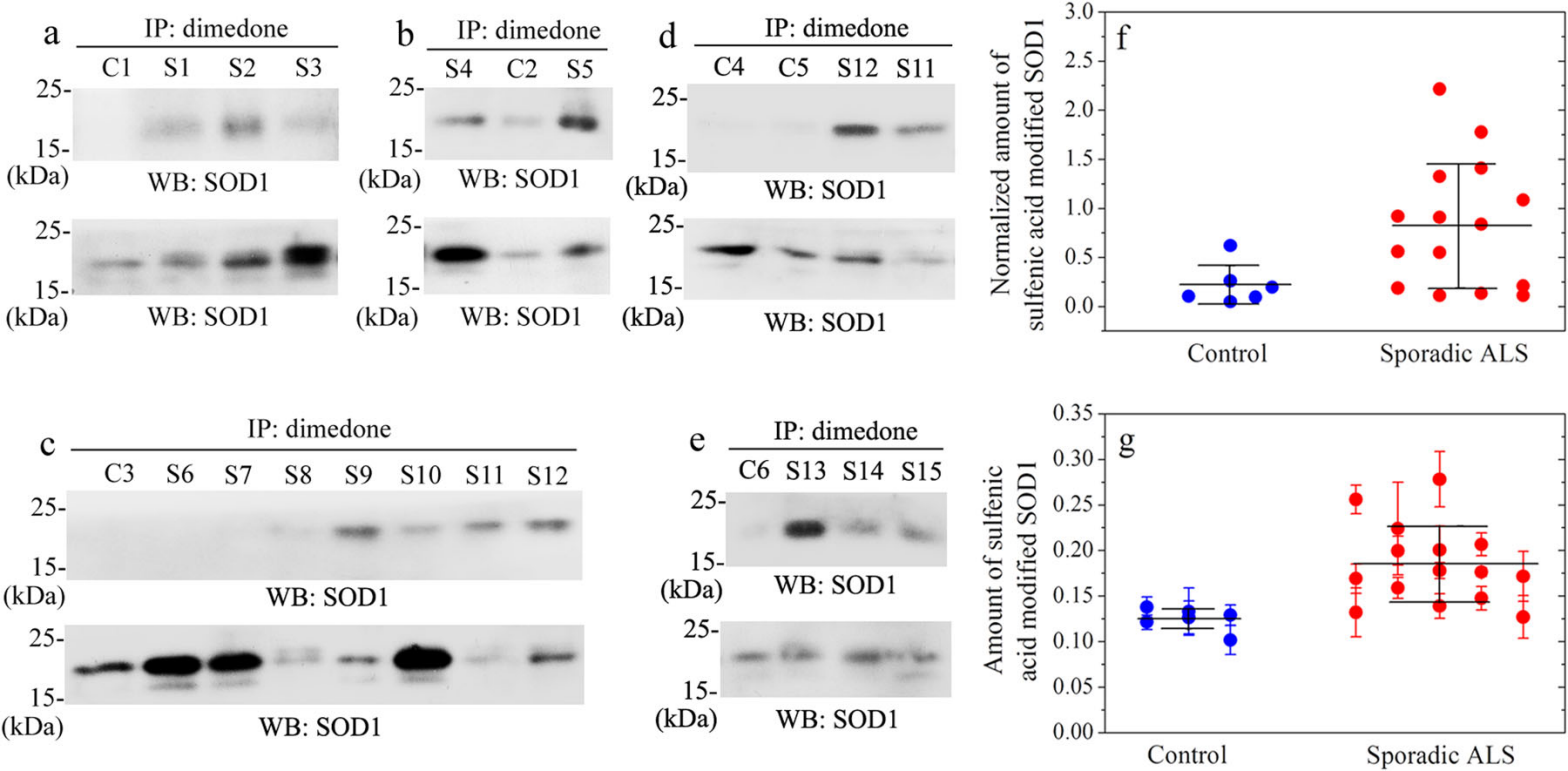
Sulfenic acid-modified wild-type SOD1 level in cerebrospinal fluid of 15 sporadic ALS patients is significantly increased compared with 6 age-matched control patients (**f**: Western blot, *F* = 0.0207 and *p* = 0.021; **g**: ELISA, *F* = 0.0126 and *p* = 0.0019) Two hundred microlitres of CSF samples were mixed with 2 μl of a cocktail protease inhibitors and 2 μl of 100 mM dimedone, immunoprecipitated with 1 μl of 1.0 mg/ml rabbit anti-dimedone polyclonal antibody overnight at 4 °C, and then incubated with 20 μl of Protein G Agarose beads for 12–14 h at 4 °C. The beads were washed thrice with PBS buffer, boiled in SDS-PAGE loading buffer without reducing agents for 5 min, and then probed with Western blot using mouse anti-SOD1 antibody (WB: SOD1, the upper lane (**a**–**e**)). Another 20 μl of CSF samples were boiled in SDS-PAGE loading buffer, probed with Western blot using rabbit anti-SOD1 antibody, and served as the input control (WB: SOD1, the lower lane (**a**–**e**)), which represented the total SOD1 content in CSF samples. Sulfenic acid modification of wild-type SOD1 was remarkably observed in CSF samples of 13 patients with sporadic ALS (S1–S5 and S8–S15, the upper lane (**a**–**e**)), but was not remarkably observed in those of 6 control patients (non-ALS patients) (C1-C6, the upper lane (**a**–**e**)) and 2 sporadic ALS patients (S6 and S7, the upper lane, **c**). Normalized amount of sulfenic acid-modified wild-type SOD1 in CSF samples of 15 patients with sporadic ALS (red solid circle, **f**) and 6 age-matched non-ALS control patients (blue solid circle, **f**) was calculated by the densitometry of sulfenic acid-modified wild-type SOD1 bands (WB: SOD1, the upper lane (**a**–**e**)), divided by that of the total SOD1 bands (WB: SOD1, the lower lane (**a**–**e**)). 100 μl of CSF samples were mixed with 1 μl of a cocktail of protease inhibitors and 1 μl of 100 mM dimedone, and added into each well of the ELISA plates coated with mouse anti-SOD1 antibody. The ELISA plates were incubated with 100 μl/well of 0.5 μg/ml rabbit anti-dimedone antibody followed by 100 μl/well of 1/10000 homologous goat anti-rabbit secondary antibody conjugated with horseradish peroxidase. Then 200 μl/well of the substrate 3,3′,5,5′-tetramethylbenzidine was added and the absorbance of the blue product was measured with a microplate reader at 630 nm. Amount of sulfenic acid-modified wild-type SOD1 in CSF samples of 15 sporadic ALS patients (red solid circle, **g**) and 6 age-matched non-ALS control patients (blue solid circle, **g**) was represented by the absorbance at 630 nm in each well. Data on absorbance with error bars were expressed as mean ± S.D. of 3 independent experiments (**g**). Statistical analyses were done using *t*-test. Values of *p* < 0.05 and *p* < 0.001 are considered as statistically significant and much significant, respectively

As mentioned above, we present an interesting observation on H_2_O_2_ dependent aggregation of wild-type SOD1, and identification of sulfenic acid modification of Cys-111 is indeed quite remarkable, we wonder whether this form of post-translational modification could be detected in neurons and CSF of ALS patients. We thus determined the amount, by Western blot (Fig. 7a–e) and ELISA (Fig. 7g) methods, of sulfenic acid-modified wild-type SOD1 in CSF from 15 patients with sporadic ALS (ages 42–63, mean ± SD 50.3 ± 6.0) and 6 age-matched non-ALS control patients (ages 50–64, mean ± SD 53.5 ± 4.8). As shown in Fig. 7a–e, sulfenic acid modification of wild-type SOD1 was remarkably observed in CSF samples from 13 patients with sporadic ALS (S1–S5 and S8–S15), but was not remarkably observed in those of 6 non-ALS control patients (C1-C6) and 2 sporadic ALS patients (S6 and S7). We found that CSF samples from sporadic ALS patients did contain significantly higher levels of sulfenic acid-modified wild-type SOD1 compared to age-matched controls (0.822 ± 0.629 in sporadic ALS versus 0.220 ± 0.192 in controls, *p* = 0.021) (Fig. 7f). We also observed a significant increase of sulfenic acid-modified wild-type SOD1 level in CSF of sporadic ALS patients, compared to age-matched controls (0.185 ± 0.042 in sporadic ALS versus 0.125 ± 0.012 in controls, *p* = 0.0019) (Fig. 7g). Furthermore, the total SOD1 level in CSF of 15 sporadic ALS patients was not significantly increased compared with 6 age-matched controls (1.80 ± 1.36 in sporadic ALS versus 0.923 ± 0.455 in controls, *p* = 0.079) (Figure S6).

Taken together, our data demonstrated that sulfenic acid-modified wild-type SOD1 level in CSF of 15 patients with sporadic ALS was significantly increased compared to 6 age-matched control patients, suggesting a role of sulfenic acid-modified wild-type SOD1 as a diagnostic biomarker in sporadic ALS.

## Discussion

In this paper, we want to answer the question whether and how H_2_O_2_ at pathological concentrations regulates the fibrillization and cytotoxicity of SOD1 and TDP-43 for two reasons. Firstly, pathological concentrations of H_2_O_2_ could cause oxidative modifications to cellular proteins including SOD1^17,29,43,44^. Secondly, the antioxidant function of SOD1^43^ exposes SOD1 to serious oxidative stress resulting from the accumulation of neurotoxic ROS during aging, and oxidative modifications to SOD1 is further enhanced by long half-life of SOD1 in motor neurons^43,45–47^. In this study, we proved that H_2_O_2_ at pathological concentrations did trigger wild-type SOD1 fibrillization via sulfenic acid modification of SOD1. We also demonstrated that the sulfenic acid-modified SOD1 oligomers caused TDP-43 redistribution from the nuclei to the cytoplasm, an ongoing event during ALS progression^48^, and induced SOD1/TDP-43 to form amyloid fibrils in cytoplasm of neuronal cells. In other words, fibrils composed of SOD1 coexisted with those composed of TDP-43 in cells. Interestingly, the accumulation of pathological TDP-43 has been found to coexist with misfolded SOD1 in patient motor neurons^7,19^. Our data establish a novel role for pathological H_2_O_2_, whose concentration increases with age^43,47^, in regulating the misfolding and toxicity of SOD1 and TDP-43.

The Valentine lab has pioneered a study of SOD1 fibril formation under physiological reducing conditions^3,22^, and the Eisenberg lab has reported a structure for toxic SOD1 oligomers^49^. Here, we firstly reported a study of SOD1 fibril formation under pathological oxidative conditions, which are closer to the pathological environments in ALS and FTLD patient’s brain and spinal cord^11^. By using anti-dimedone antibody, we found that Cys-111 in wild-type SOD1 was oxidized to C-SOH by pathological H_2_O_2_, and demonstrated that a small amount of sulfenic acid-modified SOD1 oligomers did induce the fibrillization and toxicity of SOD1 and TDP-43 in neuronal cells. By using AFM, we observed a mixture of some long amyloid fibrils and abundant spherical or ellipsoidal particles formed by apo-SOD1 when treated with pathological H_2_O_2_. Interestingly, inclusions observed in ALS mouse models and patients are composed of some thick fibrils and spherical or ellipsoidal particles^45^. We thus suggest that post-translational modifications to wild-type SOD1 should play significant roles in the misfolding and cytotoxicity of SOD1 and TDP-43, based on this work and the reported results^12,17,50^. In this study, CSF from ALS patients was chosen for investigation because of the following reasons. Firstly, CSF is a proximal fluid to the site of ALS onset, giving CSF advantages for diagnostic biomarker discovery over more distal fluids including plasma^51^. Secondly, obtaining CSF samples via lumbar puncture is much safer and more practical than biopsy of the brain or spinal cord from ALS patients^52^. Thirdly, obtaining autopsy brain samples from ALS patients is quite difficult in China. ALS and FTLD patients have a significant increase of TDP-43 level in their CSF compared to age-matched controls^53,54^. It has been reported that total SOD1 and misfolded SOD1 in CSF cannot be used as biomarkers for sporadic ALS^52,55^, and it is controversial whether misfolded SOD1 is accumulated in spinal cords and lymphocytes from sporadic ALS patients^3,12,17,23,56^. Therefore, specific SOD1 post-translational modifications may be an important biomarker for sporadic ALS. In the current study, we reported for the first time that CSF sulfenic acid-modified wild-type SOD1 level was significantly increased in 15 patients with sporadic ALS as compared with 6 age-matched control patients, but these ALS patients did not have significantly greater levels of total SOD1 in their CSF than age-matched controls. Moreover, the level of sulfenic acid-modified wild-type SOD1 in CSF could be determined in living ALS patients. A few biomarkers for ALS, such as phosphorylated neurofilament heavy chain and TDP-43 in CSF, have been reported^52–54,57–59^. Our findings suggest that the quantification of sulfenic acid-modified SOD1 in CSF could have potential value as a diagnostic laboratory tool for sporadic ALS patients, and suggest a role of sulfenic acid-modified wild-type SOD1 as a diagnostic biomarker in sporadic ALS.

Wild-type human SOD1 has four cysteine residues, two of which (Cys-111 and Cys-6) are present as free cysteines^31,60,61^. Cys-111, the most solvent-exposed Cys in human SOD1, is a highly reactive Cys placed on the surface, whereas Cys-6 is deeply buried inside this enzyme^31,60,61^. It has been reported that Cys-111 is a key residue for the aggregation and cytotoxicity of human SOD1 pathogenic mutants^62–66^. The major finding of our study is that H_2_O_2_ at pathological concentrations (as low as 20 μM) did trigger the fibrillization of wild-type human SOD1 via sulfenic acid modification of Cys-111 and subsequently induced SOD1/TDP-43 toxicity in neuronal cells. Therefore, Cys-111 is a key residue for the misfolding and cytotoxicity of wild-type SOD1 kindled by pathological H_2_O_2_, and the sulfenation of Cys-111 in SOD1 could perform a pivotal function in the pathogenesis of sporadic ALS and FTLD.

**Fig. 8:**
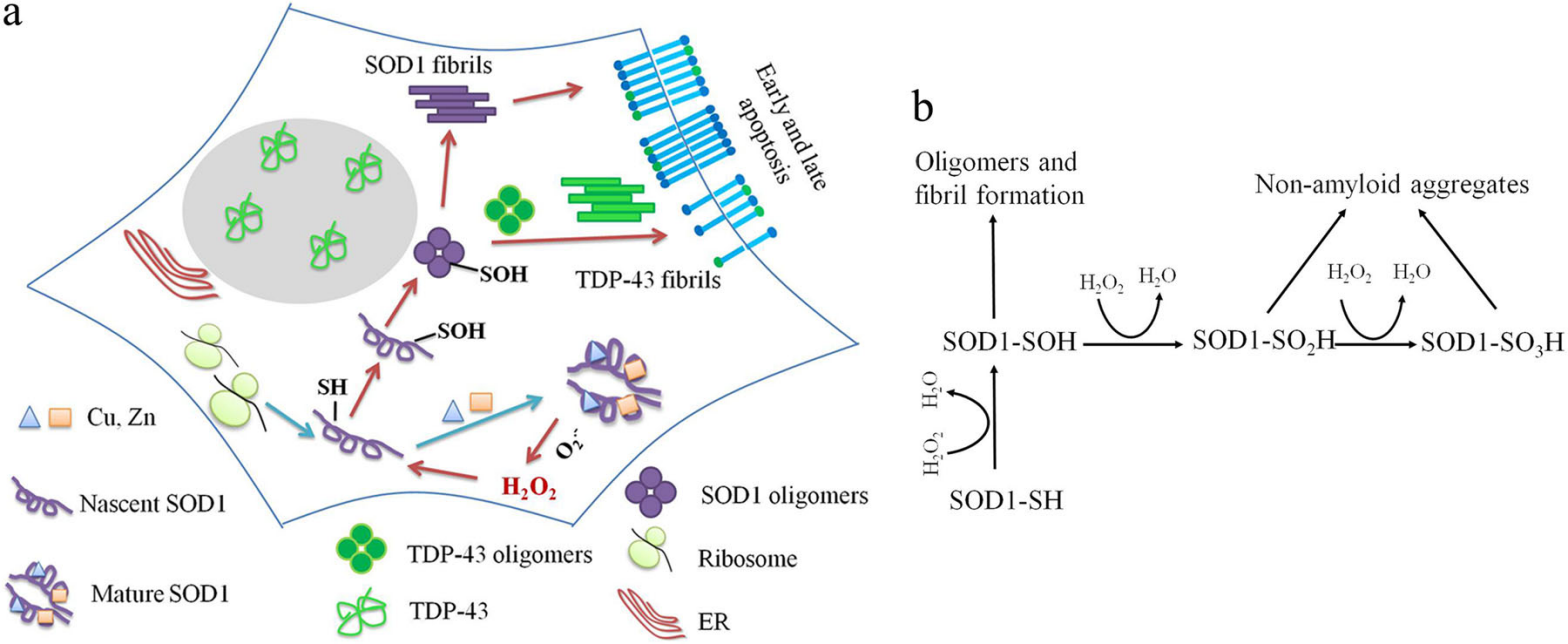
A hypothetical model shows how pathologic concentration of hydrogen peroxide triggers the misfolding of SOD1 via sulfenic acid modification of Cys-111, and thus induces SOD1 toxicity and TDP-43 toxicity in neuronal cells Nascent SOD1 (purple unfolded monomer, apo-SOD1) overexpressed in SH-SY5Y cells emerges from the ribosome (light green), copper (blue triangle), and zinc (orange square) bind to the correct binding sites, and the disulfide bond and mature SOD1 (purple dimer) are formed step by step (**a**). Mature SOD1 catalyzes the dismutation of superoxide anion (${\mathrm{O}}_{\mathrm{2}}^{ \bullet - }$) to H_2_O_2_ and O_2_, and pathological concentration of the product H_2_O_2_ (red) abnormally accumulated in cytoplasm of SH-SY5Y cells could then trigger the oligomerization and fibrillization of nascent SOD1 via sulfenic acid modification of Cys-111 (from SOD1-SH to SOD1-SOH, purple), forming sulfenic acid-modified SOD1 oligomers (purple balls) and amyloid fibrils (purple bars) in SH-SY5Y cells (**a**). Sulfenic acid-modified SOD1 oligomers could induce cytoplasm mislocalization of human TDP-43 (green) (from the nucleus to the cytosol), forming cytoplasmic TDP-43 oligomers (green balls) and cytoplasmic fibrils (green bars), and induce apoptosis of living SH-SY5Y cells, thereby increasing the cytotoxicity of SOD1 and TDP-43 (**a**). Phosphatidylserine (**a**) is indicated by blue sticks with green balls. Scheme describing the competition between the major pathway, in which pathological concentration of H_2_O_2_ oxidizes human SOD1 to SOD1-SOH and drives human SOD1 to form highly toxic oligomers and fibrils, and another pathway, in which H_2_O_2_ at high concentrations over-oxidizes human SOD1 to SOD1-SO_2_H and SOD1-SO_3_H and drives human SOD1 to form non-amyloid aggregates (**b**)

Figure 8 shows a hypothetical model on how H_2_O_2_ triggers SOD1 fibrillization via sulfenic acid modification of Cys-111. Mature SOD1 catalyzes the disproportionation of ${\mathrm{O}}_{\mathrm{2}}^{ \bullet - }$ to H_2_O_2_ and O_2_, and pathological H_2_O_2_ abnormally accumulated in the cytoplasm could then trigger nascent SOD1 aggregation via sulfenation of Cys-111, forming sulfenic acid-modified SOD1 oligomers and fibrils in neuronal cells. Sulfenic acid-modified SOD1 oligomers could induce TDP-43 cytoplasm localization (mistakenly from the nuclei to the cytoplasm), forming cytoplasmic TDP-43 oligomers and fibrils, and induce early and late apoptosis of neuronal cells, thereby increasing the cytotoxicity of SOD1 and TDP-43. The above model can explain how H_2_O_2_ at pathological concentrations regulates the misfolding and toxicity of SOD1 and TDP-43 in the ALS brain and spinal cord. The inhibition of wild-type SOD1 aggregation by dimedone (this work) and the inhibition of mutant SOD1 aggregation by macrophage migration inhibitory factor^67^ provide a feasible method for treatment of sporadic ALS and familial ALS, respectively.

## Materials and Methods

### Materials

Three dyes, ThT, ThS, and DAPI, and four antibodies, mouse anti-FLAG monoclonal antibody, rabbit anti-FLAG monoclonal antibody, 10 nm gold-labeled anti-mouse antibody, and mouse anti-β-tubulin antibody, were bought from Sigma-Aldrich (St. Louis, MO). Sarkosyl and Triton X-100 were purchased from Amresco (Solon, OH). Rabbit anti-SOD1 polyclonal antibody, mouse anti-SOD1 monoclonal antibody, and 10 nm gold-labeled anti-rabbit antibody were from Abcam (Cambridge, MA). Streptavidin, DyLight-405 conjugated secondary antibody and secondary antibody conjugated with Alexa 546/488 were from Invitrogen (Carlsbad, CA). Anti-dimedone polyclonal antibody was purchased from Millipore (Billerica, MA). Other chemicals were analytical grade, made in China.

### SOD1 expression and purification

Single cysteine mutant C111S was generated by using primers TCTCAGGAGACCATAGCATCATTGGCC/ GGCCAATGATGCTATGGTCTCCTGAGA. The expression, purification, demetalization, and concentration determination of wild-type SOD1 and C111S were the same as described previously^3,68^. AAnalyst-800 atomic absorption spectrometer (PerkinElmer) was used to quantify metal content of SOD1 samples. Samples of wild-type SOD1 and C111S contained less than 5% of residual metal ions, indicating that the samples were indeed in the apo state.

### Cell culture

SH-SY5Y/HEK293T cells were cultured and HEK293T cells were transiently transfected with FLAG-tagged wild-type SOD1 or its C111S variant in pCMV-Tag 2B vector as described^37,38^. SOD1 stable cells and TDP-43 stable cells were constructed and cultured according to previously published protocols^37,38^. All the below experiments were repeated 3 times.

### ThT binding assays

A 2.5 mM ThT fresh solution was prepared in 20 mM Tris-HCl buffer at pH 7.4 as described^33^. Thirty micrometer apo wild-type SOD1 was incubated without or with 20–200 μM H_2_O_2_ for up to 45 h. Thirty micrometer apo-SOD1 was incubated with 50 μM H_2_O_2_, but without dimedone and then titrated with 50–500 μM dimedone at 0 h or 200 μM dimedone at 32 h, respectively, or incubated with 100 μM H_2_O_2_, but without dimedone and then titrated with 1.0 mM dimedone. Fifty microlitre of SOD1 samples and 5.0 μl of ThT solution were diluted into 20 mM Tris-HCl buffer, giving an ultimate volume of 0.50 ml and final concentrations of 3 and 25 μM for apo wild-type SOD1 and ThT, respectively. LS-55 luminescence spectrometer (Perkin-Elmer) was used to measure ThT fluorescence excited at 440 nm and emitted at 480 nm with slit widths of 10.0 and 5.0 nm, respectively. Kinetic experiments were repeated 3 times at 37 °C with agitation. The determination of kinetic parameters of SOD1 fibrillization was made according to a sigmoidal equation^3,33,34^.

### Circular dichroism spectroscopy

Under standard conditions, 30 μM apo wild-type SOD1 was incubated without or with 20–200 μM H_2_O_2_ in 20 mM Tris-HCl buffer at pH 7.4 and 37 °C for 45 h or 20 h. Jasco J-810 spectropolarimeter (Jasco Corp., Tokyo, Japan) was used to obtain far-UV CD spectra of the fibrillization 10 μM SOD1. The mean residue molecular weight for SOD1 is 104.6 Da. The mean residue molar ellipticity (*θ*) was determined by the equation described^69^.

### TEM

Formation of fibrils by apo wild-type SOD1 incubated with 20–200 μM H_2_O_2_ or non-amyloid aggregates by this protein incubated with 1.0–2.0 mM H_2_O_2_ was confirmed by electron microscopy of negatively stained samples. Sample preparation and TEM analysis were described in detail previously^33^.

### AFM

AFM was used to probe the structural order in the aggregates produced from 30 μM apo wild-type SOD1 incubated with 100 μM H_2_O_2_ with the aim to see if these are still fibrils or disordered complexes. Sample preparation and AFM analysis were described in detail previously^33^.

### Laser scanning confocal analysis

To confirm fibril formation of wild-type SOD1 in cells triggered by pathological concentration of H_2_O_2_ or induced by sulfenic acid-modified SOD1 oligomers, SOD1 stable cells and SH-SY5Y cells were cultured in medium containing 0–100 μM H_2_O_2_ for 4 and 6 days, respectively, or grown in medium incubated with 0–5 μM sulfenic acid-modified SOD1 oligomers or 0–1 μM biotinylated sulfenic acid-modified SOD1 oligomers for 3 days, then fixed and ruptured. The cells were stained with ThS or streptavidin DyLight-405 dye, and coimmunostained with mouse anti-FLAG antibody or rabbit anti-SOD1 antibody and homologous secondary antibody conjugated with Alexa Fluo-546.

To observe cytoplasm mislocalization and the subsequent fibrillization of wild-type TDP-43 in cells induced by sulfenic acid-modified SOD1 oligomers, TDP-43 stable cells were grown in medium incubated with 0–5 μM sulfenic acid-modified SOD1 oligomers for 3 days, then fixed and ruptured. The cells were coimmunostained with mouse primary anti-FLAG antibody and homologous secondary antibody conjugated with Alexa Fluo-488. The nuclei were stained with DAPI.

Sample preparation and laser scanning confocal analysis were described in detail previously^38^.

### Super-resolution fluorescence microscopy

To clearly observe cytoplasm mislocalization and the subsequent fibrillization of wild-type TDP-43 in cells induced by sulfenic acid-modified SOD1 oligomers, TDP-43 stable cells were grown in medium incubated with 0-5 μM sulfenic acid-modified SOD1 oligomers for 3 days, then fixed and ruptured. The cells were immunostained with primary mouse anti-FLAG antibody and homologous secondary antibody conjugated with Alexa Fluo-488, and then immunostained with primary rabbit anti-SOD1 antibody and secondary donkey anti-rabbit IgG conjugated with Alexa Fluo-546. The nuclei were stained with DAPI. Sample preparation and super-resolution fluorescence analysis were described in detail previously^38^.

### Western blot

SH-SY5Y cells and SOD1 stable cells were grown in medium incubated with 0–100 μM H_2_O_2_ for 6 and 4 days, respectively, or grown in medium incubated with 0–5 μM sulfenic acid-modified SOD1 oligomers for 3 days, then harvested, lysed, and centrifuged. The supernatants were incubated with Sarkosyl, and the Sarkosyl-insoluble proteins were acquired by ultracentrifugation. Such samples were subjected to SDS-PAGE and transferred onto the membranes, and blocked with 5% fat-free milk in 100 mM Tris-HCl buffer containing 150 mM NaCl and 0.1% Tween 20. The membranes were incubated with mouse anti-FLAG antibody, 1/2000 rabbit anti-SOD1 antibody or mouse anti-β-actin antibody, and then 1/4000 secondary antibody conjugated with horseradish peroxidase. The protocol for Western blot was described in detail previously^38^.

### Immunoelectron microscopy

SOD1 stable cells and SH-SY5Y cells were cultured in medium containing 0–100 μM H_2_O_2_ for 4 and 6 days, respectively, and SOD1/TDP-43 stable cells were cultured in medium containing 0–5 μM sulfenic acid-modified SOD1 oligomers for 3 days. The cells were homogenized and centrifuged at 17,000 g for 20 min. After centrifugation of the supernatants at 150,000 g for 30 min, the Sarkosyl-insoluble pellets were resuspended in PBS buffer. Sample aliquots of 10 μl were absorbed onto nickel grids and incubated with 1/300 mouse anti-FLAG antibody or rabbit anti-SOD1 antibody. After blocked with BSA, 10-nm gold-labeled homologous secondary antibodies were used to incubate the grids. The protocol for immunoelectron microscopy was described in detail previously^37,38^.

### Sulfenic acid modification of wild-type SOD1 in vitro

Ten micrometer of apo wild-type SOD1 and its cysteine mutant C111S were incubated with 0–200 μM H_2_O_2_ or 0–10 mM H_2_O_2_ for 30 min at 25 °C, and blocked with 5 mM dimedone. The proteins were subjected to 13.5% SDS-PAGE and transferred onto the membranes (Millipore), and blocked with 5% fat-free milk in 50 mM Tris-HCl buffer containing 150 mM NaCl and 0.05% Tween 20. The membranes were incubated with 1/1000 anti-FLAG antibody or 1/1000 anti-dimedone antibody, and then 1/5000 secondary antibody conjugated with horseradish peroxidase. Western blot was developed using Immobilon^TM^ Western Horseradish Peroxidase Substrate Luminal Reagent (Millipore) and visualized using a Chemiluminescence Imaging System (Clinx Science Instruments, Shanghai, China).

### Sulfenic acid modification of wild-type SOD1 in cells

HEK293T cells overexpressing wild-type SOD1 and its C111S variant with Flag tag transiently were incubated with 0–100 μM H_2_O_2_ at 25 °C for 2 h, and blocked with 5 mM dimedone. After incubation, the cells were harvested, washed with PBS buffer for three times, and lysed in 200 µl ice-cold RIPA buffer with protease inhibitor cocktails. The supernatant was gathered and the protein concentrations of the cellular extracts were determined by BCA assay (Beyotime, Nantong, China). *α*-Flag M2 affinity gel (Sigma-Aldrich) was used to immunoprecipitate Flag-tagged SOD1. Immunoprecipitation was performed at 4 °C and in RIPA buffer with protease inhibitor cocktails overnight. The beads were then washed for 3 times with PBS buffer, and the resuspended samples were detected by Western blot using anti-dimedone or anti-FLAG antibodies.

### Nano-LC-MS/MS analysis

The Coomassie Blue-stained gels of 10 μM apo wild-type SOD1 treated with 100 μM H_2_O_2_ for 30 min and blocked with 5 mM dimedone were scissored out, chopped, trypsinized, and then analyzed with nano-LC-MS/MS. The tandem mass spectrometric experiments were carried out on a Q Exactive Orbitrap LC-MS/MS System (Thermo Scientific, Waltham, MA). The segmented gradient was 5–8% Solvent B, 8 min; 8–22% Solvent B, 50 min; 22–32% Solvent B, 12 min; 32–95% Solvent B, 1 min; and 95% Solvent B, 7 min. The normalized collision energy for HCD and the dynamic exclusion time were 27% and 40 s, respectively. The protocol for nano-LC-MS/MS analysis was described in detail previously.^70^

The Coomassie Blue-stained gels of 30 μM apo wild-type SOD1 treated with 10.0 mM H_2_O_2_ for 30 min were scissored out, chopped, trypsinized, but without reduction/alkylation of cysteines, and then analyzed with nano-LC-MS/MS. The tandem mass spectrometric experiments were carried out on a Q Exactive HF Orbitrap LC-MS/MS System (Thermo Scientific). A 25 cm Acclaim PepMap C18 column with a 60-min gradient was used to separate the peptides. Q Exactive HF was operated in data-dependent acquisition mode with repeated full MS scan (R = 120 K, AGC = 3e6, maximum injection time = 20 ms, scan range = 300–1800 m/z) followed by twenty MS/MS scans (R = 60 K, AGC = 1e6, maximum injection time = 105 ms). Proteome Discovery version 2.1 and Sequest HT search engine for protein identification were employed to analyze the raw data from Q Exactive HF, against a human SwissProt protein database.

Some important searching parameters were set as following: trypsin was selected as enzyme; the mass tolerance of precursor and the product ions tolerance were 10 ppm and 0.02 Da, respectively; dimedone labeling and carbamidomethylation of cysteines were selected as variable modifications for mass spectrometric experiments to detect sulfenic acid modification of SOD1; and dioxidation and trioxidation of cysteine, cysteine oxidation, and deamidation of asparagine and glutamine were selected as variable modifications for mass spectrometric experiments to detect sulfinic/sulfonic acid modification of SOD1.

### Annexin V-FITC apoptosis detection assay

SOD1 stable cells and TDP-43 stable cells were cultured in medium containing 5 μM sulfenic acid-modified SOD1 oligomers for 3 days, and then double stained with annexin V-FITC and PI. Olympus FluoView FV1000 laser scanning confocal microscope (Tokyo, Japan) was used to observe apoptotic cells.

Flow cytometry was used to detect apoptotic cells double stained by annexin V-FITC and PI. SOD1 stable cells and TDP-43 stable cells were treated with 10 μM sulfenic acid-modified SOD1 oligomers for 3 days to prepare apoptotic cells induced by such SOD1 oligomers. The protocol for flow cytometry analysis was described in detail previously.^38^

### Cerebrospinal fluid samples

The cerebrospinal fluid samples were obtained from 15 sporadic ALS patients (ages 42–63, 50.3 ± 6.0) and 6 age-matched non-ALS control patients (ages 50–64, 53.5 ± 4.8) in the Department of Neurology, Tongji Hospital. See Table 1 for clinical details. The sporadic ALS patients examined in this correspondence were definitely diagnosed (*n* = 14) or probably diagnosed (*n* = 1), and did not have a family history of ALS. The age-matched non-ALS control subjects comprised 6 disease controls including patients with Alzheimer disease (*n* = 1), type 2 diabetes overlapped by necrotizing myopathy (*n* = 1), brainstem infarction (*n* = 1), intracranial infection (*n* = 1), Guillain–Barré syndrome (*n* = 1), and cerebral cysticercosis (*n* = 1). Fresh cerebrospinal fluid samples were collected from living sporadic ALS and control cases, and then stored at -80 °C until used for immunoprecipitation, Western blot, and ELISA.

**Table 1:** Clinical details of patients with ALS and non-ALS at the time when CSF samples were taken

Case	Gender	Age	Mean age ± S.D.	Clinical diagnosis
Non-ALS				
1	M	52	53.5 ± 4.8	Alzheimer disease
** 2**	M	50		Brainstem infarction
3	M	64		Intracranial infection
4	M	52		Type 2 diabetes overlapped by necrotizing myopathy
5	M	51		Guillain–Barré syndrome
6	M	52		Cerebral cysticercosis
Sporadic ALS				
1	M	56	50.3 ± 6.0	Sporadic ALS
2	M	49		Sporadic ALS
3	M	51		Sporadic ALS
4	M	56		Sporadic ALS
5	M	63		Sporadic ALS overlapped by Dementia with Lewy Bodies
6	M	48		Sporadic ALS
7	M	42		Sporadic ALS
8	F	57		Sporadic ALS
9	M	47		Sporadic ALS
10	M	43		Sporadic ALS
11	M	42		Sporadic ALS
12	M	45		Sporadic ALS
13	F	50		Sporadic ALS
14	F	49		Sporadic ALS
15	M	56		Sporadic ALS

*M* male, *F* female

### Immunoprecipitation (IP) and western blot

Two hundred microlitre of cerebrospinal fluid samples were mixed with 2 μl of a cocktail of protease inhibitors and 2 μl of 100 mM dimedone, immunoprecipitated with 1 μl of 1.0 mg/ml rabbit anti-dimedone antibody overnight at 4 °C, and then incubated with 20 μl of Protein G Agarose beads for 12–14 h at 4 °C. The beads were washed thrice with PBS buffer, boiled in SDS-PAGE loading buffer without reducing agents for 5 min, and then probed with Western blot using mouse anti-SOD1 antibody. Another 20 μl of CSF samples were boiled in SDS-PAGE loading buffer and then probed with Western blot using rabbit anti-SOD1 antibody and served as the input control. Normalized amount of sulfenic acid-modified SOD1 in CSF samples of 15 patients with sporadic ALS and 6 age-matched non-ALS control patients was calculated by the densitometry of sulfenic acid-modified SOD1 bands divided by that of the total SOD1 bands.

### ELISA protocol

ELISA plates were coated by overnight incubation at 4 °C with 0.5 μg/ml mouse anti-SOD1 antibody, 100 μl /well, diluted in 200 mM NaHCO_3_ buffer (pH 9.6). The plates were washed thrice with PBST buffer, and blocked with 100 μl/well of PBST buffer containing 5% fat-free milk at 37 °C for 2 h. Hundred microlitre of CSF samples were mixed with 1 μl of a cocktail of protease inhibitors and 1 μl of 100 mM dimedone, and added into each well of the plates. The ELISA plates were treated with 100 μl/well of 0.5 μg/ml rabbit anti-dimedone antibody and then 100 μl/well of 1/10000 homologous goat anti-rabbit secondary antibody conjugated with horseradish peroxidase. Two hundred microlitre/well of the substrate 3,3′,5,5′-tetramethylbenzidine was added and the absorbance of the blue product 3,3′,5,5′-tetramethyl-4,4′-diphenoquinone was measured with a microplate reader at 630 nm. Statistical analyses were done using *t*-test with three times repeats. Values of *p* < 0.05 or *p* < 0.001 are considered as statistically significant or much significant.

### Ethics statement

The Institutional Review Board of the College of Life Sciences (CLS), Wuhan University, headed by Dr. Bao-Liang Song, authorized all research involving original human work, in accordance with the guidelines for the protection of human subjects. This study was authorized by the ethics committee of CLS, Wuhan University, leaded also by Prof. Song, the Dean of the college.

## Electronic supplementary material

Supplemental Data
