# Supplementary material for: Pathological hydrogen peroxide triggers the fibrillization of wild-type SOD1 via sulfenic acid modification of Cys-111
Source: Cell Death Dis. 2018 Jan 22;9(2):67. doi: 10.1038/s41419-017-0106-4 (PMC5833406; doi:10.1038/s41419-017-0106-4)
Supplement: Supplementary file 1 — Supplemental Data [file 41419_2017_106_MOESM1_ESM.pdf]

Pathological hydrogen peroxide triggers the fibrillization of wild-type SOD1 via sulfenic acid modification of Cys-111

Wen-Chang Xu[1], Jin-Zhao Liang[2,3], Cheng Li[1], Zhi-Xin He[1], Han-Ye Yuan[1], Ben-Yan Huang[4], Xiao-Ling Liu[1], Bo Tang[5], Dai-Wen Pang[5], Hai-Ning Du[6], Yi Yang[7], Jie Chen[1], Lei Wang[2,3], Min Zhang[*,4] and Yi Liang[*,1]

[1]State Key Laboratory of Virology, College of Life Sciences, Wuhan University, Wuhan 430072, China; [2]National Laboratory of Biomacromolecules, CAS Center for Excellence in Biomacromolecules, Institute of Biophysics, Chinese Academy of Sciences, Beijing 100101, China; [3]University of Chinese Academy of Sciences, Beijing 100049, China; [4]Department of Neurology, Tongji Hospital, Tongji Medical College, Huazhong University of Science and Technology, Wuhan 430030, China; [5]College of Chemistry and Molecular Sciences, State Key Laboratory of Virology, Wuhan University, Wuhan 430072, China; [6]Hubei Key Laboratory of Cell Homeostasis, College of Life Sciences, Wuhan University, Wuhan 430072, China; [7]State Key Laboratory of Bioreactor Engineering, School of Pharmacy, East China University of Science and Technology, Shanghai 200237, China

*Corresponding authors: Y Liang, State Key Laboratory of Virology, College of Life Sciences, Wuhan University, Wuhan 430072, China. Tel/fax: +86 27 68754902; E-mail: liangyi@whu.edu.cn, and M Zhang, E-mail: zhang_min_3464@126.com

Supplemental Data

Figure S1. Hydrogen peroxide at high concentrations induces wild-type human SOD1 to form non-amyloid aggregates. Negative-stain TEM graphs of non-amyloid aggregates of 30 μM apo wild-type SOD1 treated with 1.0-2.0 mM H_2O_2 (**a**, 1.0 mM; and **b**, 2.0 mM) at 37°C for 72 h. The scale bars represent 200 nm.

Figure S2. Cys-111 in wild-type SOD1 is over-oxidized to C-SO_2H and C-SO_3H by H_2O_2 at high concentrations. We clearly observed over-oxidized forms of wild-type SOD1 (SOD1-SO_2H/-SO_3H) (upper shifted band) when SOD1 was treated with 1.0-10.0 mM H_2O_2 for 30 min (**a**). 13.5% SDS-PAGE of 15 μM apo-SOD1 treated with 0-10.0 mM H_2O_2 for 30 min at 25°C (**a**). H_2O_2 concentration from left to right was zero, 0.1, 1.0, 5.0, and 10.0 mM, respectively. 30 μM apo SOD1 was treated with 10.0 mM H_2O_2 for 30 min, applied to 13.5% SDS-PAGE, and then stained with Coomassie Blue R250. The upper shifted band in the gels was scissored out, chopped, trypsinized, and analyzed with LC MS/MS. H^{80}VGDLGNVTADKDGVADVSIE DSVISLSGDHCIIGR^{115}, a MS^2 analysis of the parent peptide digested by trypsin (**b** and **c**). Analysis of the y-ions indicates both sulfinic acid modification (+31.99 Da) (**b**) and sulfonic acid modification (+47.98 Da) (**c**) of Cys-111 in wild-type SOD1 by H_2O_2 at high concentrations.

Figure S3. Effect of dimedone on the fibrillization of wild-type SOD1 induced by

H_2O_2 at a low concentration. 1.0 mM dimedone added at 6 h in the lag phase almost blocked the fibrillization of apo wild-type SOD1 *in vitro*. 30 μM apo-SOD1 was treated with 100 μM H_2O_2 but without dimedone (open square) and then titrated with 1.0 mM dimedone (solid circle). Black arrows indicate the beginning of titrations. Solid lines show the best sigmoidal fit for the ThT intensity-time curves. Thioflavin T binding assays were performed at 37°C.

Figure S4. Sulfenic acid modified SOD1 oligomers induce the fibrillization of endogenous human SOD1 in neuronal cells. SH-SY5Y cells treated with 1 μM biotinylated sulfenic acid modified SOD1 oligomers (+) (**a**-**d**), compare with SH-SY5Y cells when treated without SOD1 oligomers (-) (**e**-**h**). SH-SY5Y cells were incubated with 0 μM or 1 μM biotinylated sulfenic acid modified SOD1 oligomers for 3 days at 37°C, fixed, ruptured, detected by streptavidin DyLight-405 dye (blue), subsequently immunostained by anti-SOD1 antibody and IgG conjugated with Alexa Fluo-546 (red), and observed with confocal microscopy.

Figure S5. Sulfenic acid modified SOD1 oligomers induce the fibrillization of endogenous human SOD1 in neuronal cells. SH-SY5Y cells were treated at 37°C with 0-5 μM sulfenic acid modified SOD1 oligomers for 3 days, and then probed by Western blot. The experiments were repeated 3 times (**a**-**c**). The Sarkosyl-insoluble ultracentrifugation pellets from SH-SY5Y cells (**a**-**c**) were probed by rabbit anti-SOD1 antibody, and the total cell lysates from the cells were probed by mouse

anti-β-actin antibody. The concentrations of SOD1 oligomers from left to right (**m**) were 0 and 5 μM, respectively. Untreated cells were the control (**a-c**).

Figure S6. Total wild-type SOD1 level in CSF of 15 sporadic ALS patients is not significantly increased compared with 6 age-matched control patients ($F = 0.0320$ and $p = 0.079$). Normalized amount of total SOD1 in CSF samples of 15 patients with sporadic ALS (red solid circle) and 6 age-matched non-ALS control patients (blue solid circle) was calculated by the densitometry of the total SOD1 bands (WB: SOD1, the lower lane, Fig. 7**a-e**) divided by that of the total SOD1 band of control patient C1 (WB: SOD1, the lower lane, Fig. 7**a**).

Figure S1

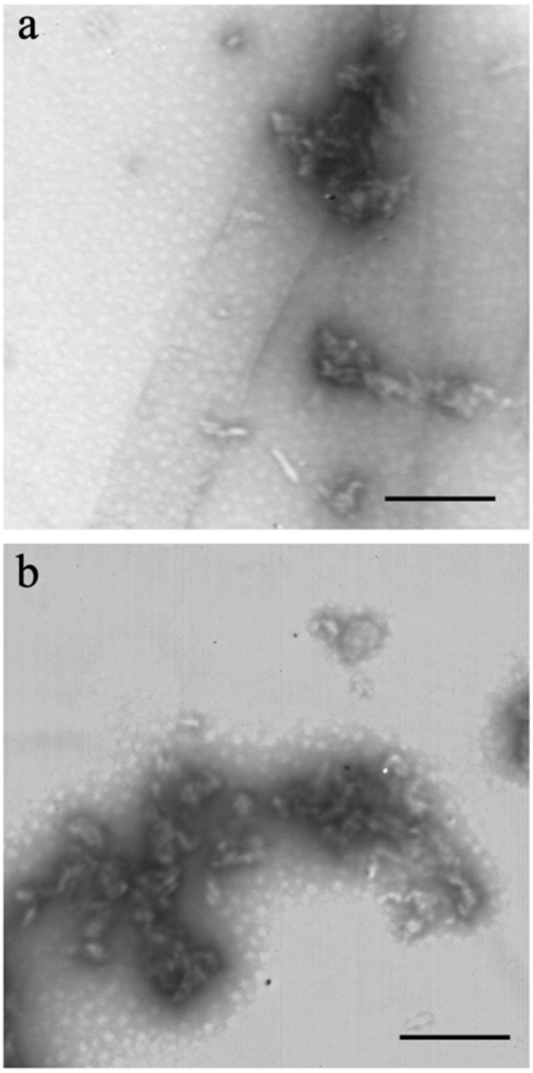

Figure S2

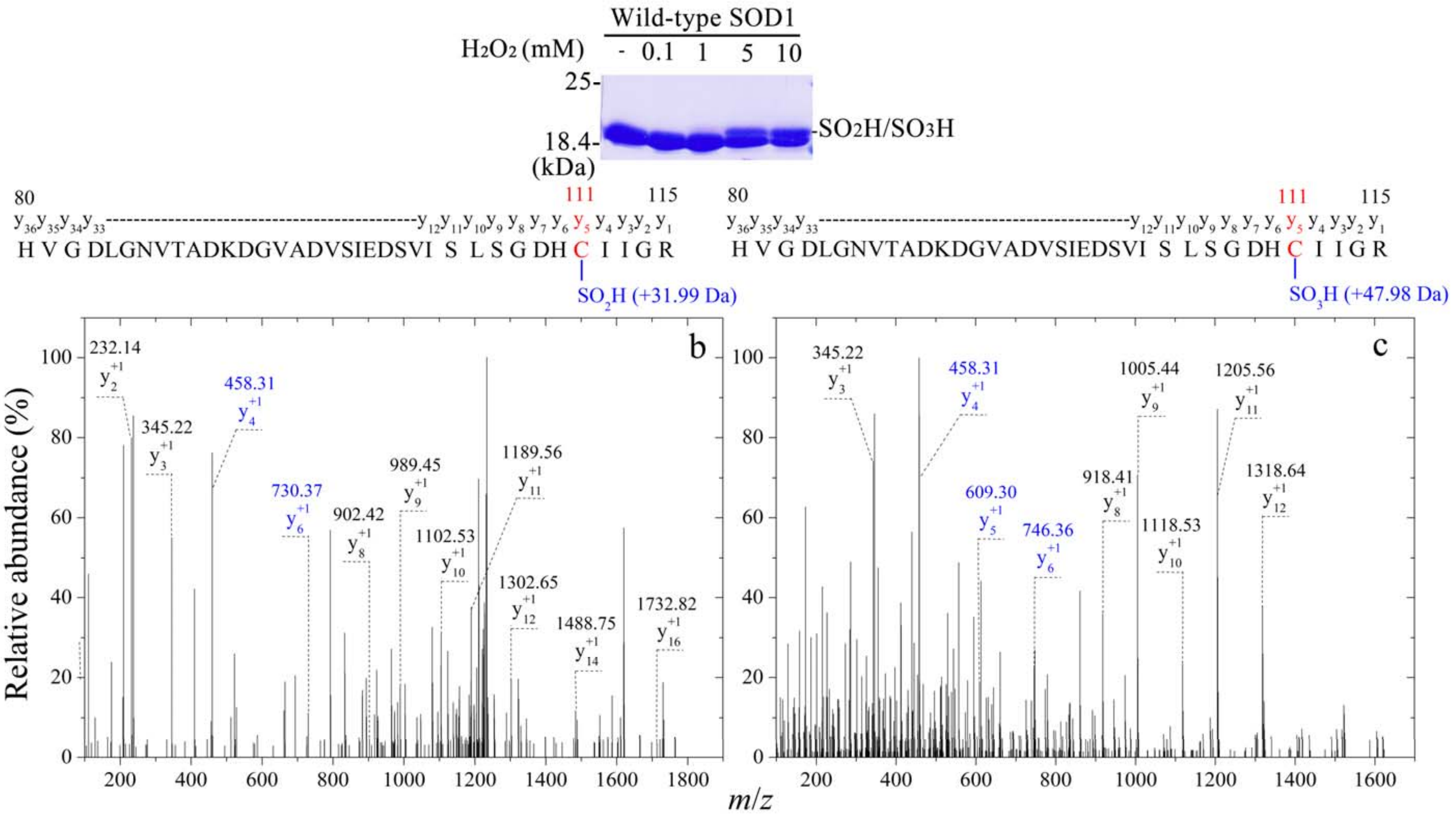

Wild-type SOD1
H2O2 (mM) - 0.1 1 5 10
25-
18.4-
(kDa)
-SO2H/SO3H
80 111 115
HVGDLGNVTADKDGVADVSIEDSVISLSGDHCIIGR
SO2H (+31.99 Da)
SO3H (+47.98 Da)
b
c
Relative abundance (%)
m/z
232.14
345.22
458.31
730.37
902.42
989.45
1102.53
1189.56
1302.65
1488.75
1732.82
609.30
746.36
918.41
1005.44
1118.53
1205.56
1318.64

Figure S3

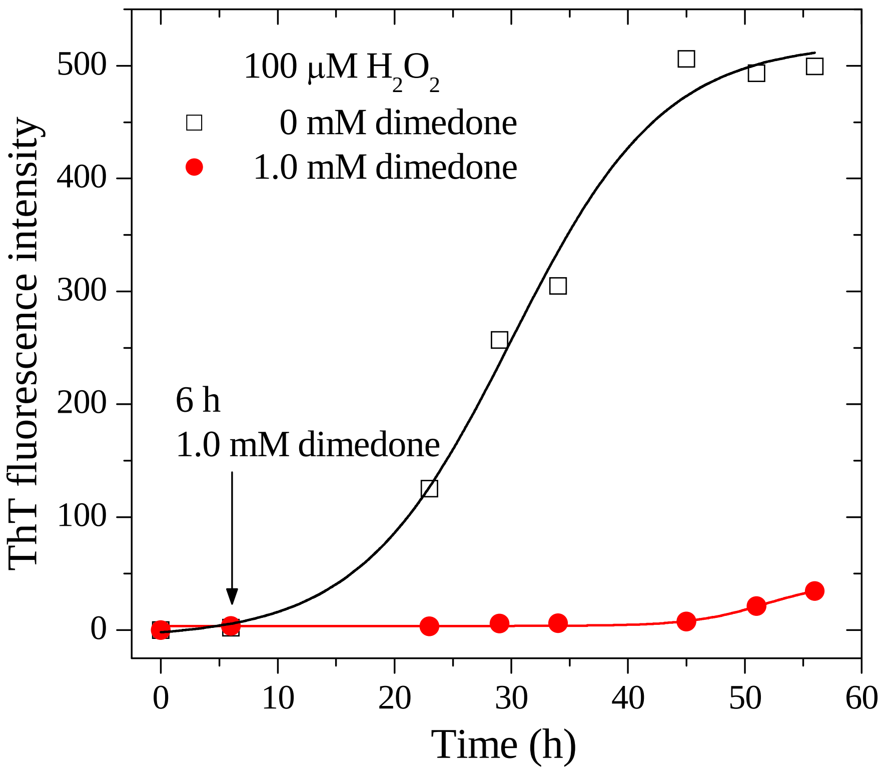
100 μM H2O2
0 mM dimedone
1.0 mM dimedone
6 h
1.0 mM dimedone
ThT fluorescence intensity
Time (h)
0
100
200
300
400
500
0
10
20
30
40
50
60

Figure S4

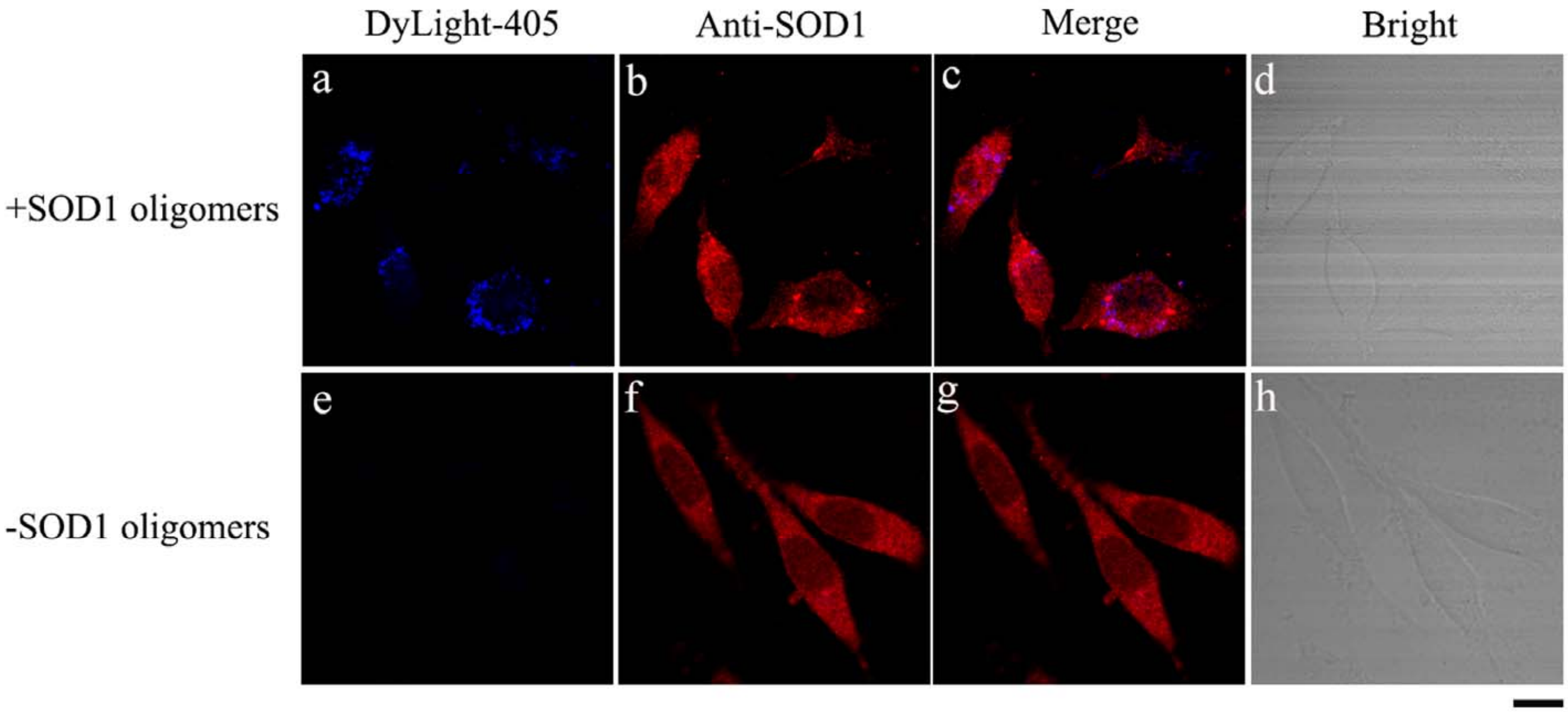
DyLight-405
Anti-SOD1
Merge
Bright
+SOD1 oligomers
-SOD1 oligomers
a
b
c
d
e
f
g
h

Figure S5

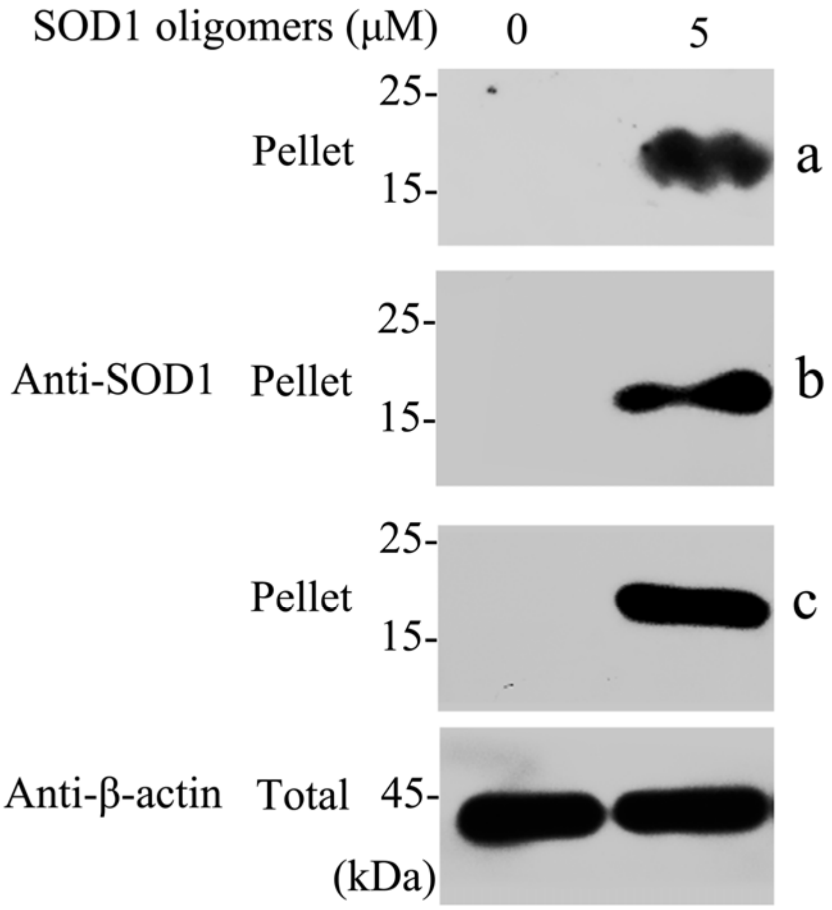
SOD1 oligomers (μM)
0
5
25-
Pellet
15-
a
25-
Anti-SOD1
Pellet
15-
b
25-
Pellet
15-
c
Anti-β-actin
Total
45-
(kDa)

Figure S6

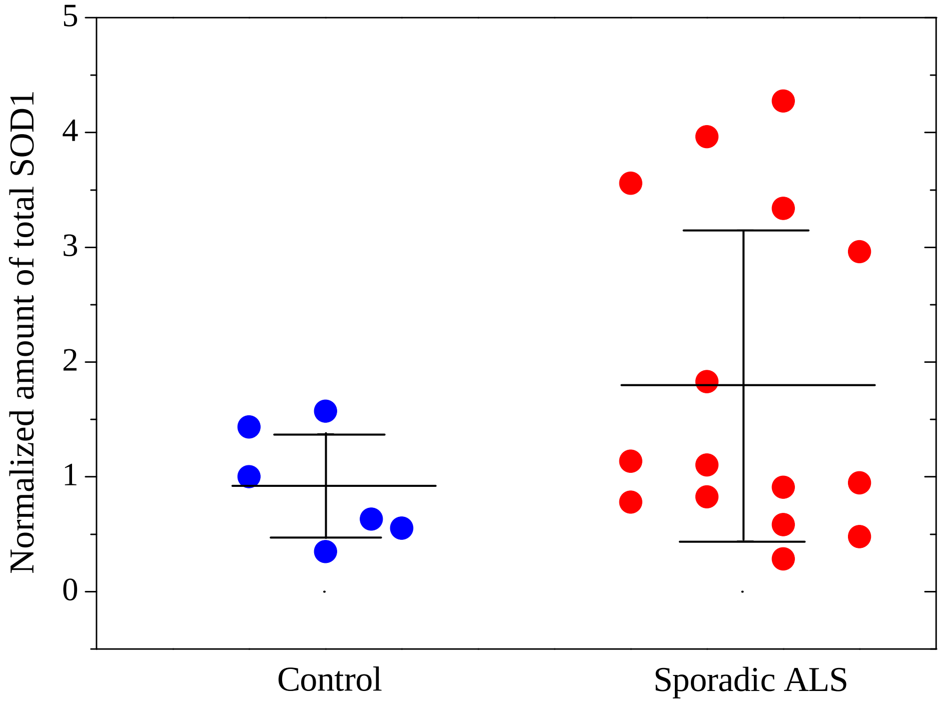
Normalized amount of total SOD1
5
4
3
2
1
0
Control
Sporadic ALS
